# HYS-32-Induced Microtubule Catastrophes in Rat Astrocytes Involves the PI3K-GSK3beta Signaling Pathway

**DOI:** 10.1371/journal.pone.0126217

**Published:** 2015-05-04

**Authors:** Chi-Ting Chiu, Chih-Kai Liao, Chien-Chang Shen, Tswen-Kei Tang, Guey-Mei Jow, Hwai-Shi Wang, Jiahn-Chun Wu

**Affiliations:** 1 Institute of Anatomy and Cell Biology, School of Medicine, National Yang-Ming University, Taipei 11221, Taiwan; 2 Department of Anatomy and Cell Biology, College of Medicine, National Taiwan University, Taipei 10051, Taiwan; 3 Division of Medicinal Chemistry, National Research Institute of Chinese Medicine, Taipei 11221, Taiwan; 4 Department of Nursing, College of Health and Nursing, National Quemoy University, Kinmen 89250, Taiwan; 5 School of Medicine, Fu-Jen Catholic University, New Taipei City 24205, Taiwan; Medical College of Wisconsin, UNITED STATES

## Abstract

HYS-32 is a novel derivative of combretastatin-A4 (CA-4) previously shown to induce microtubule coiling in rat primary astrocytes. In this study, we further investigated the signaling mechanism and EB1, a microtubule-associated end binding protein, involved in HYS-32-induced microtubule catastrophes. Confocal microscopy with double immunofluorescence staining revealed that EB1 accumulates at the growing microtubule plus ends, where they exhibit a bright comet-like staining pattern in control astrocytes. HYS-32 induced microtubule catastrophes in both a dose- and time-dependent manner and dramatically increased the distances between microtubule tips and the cell border. Treatment of HYS-32 (5 μM) eliminated EB1 localization at the microtubule plus ends and resulted in an extensive redistribution of EB1 to the microtubule lattice without affecting the β-tubulin or EB1 protein expression. Time-lapse experiments with immunoprecipitation further displayed that the association between EB-1 and β-tubulin was significantly decreased following a short-term treatment (2 h), but gradually increased in a prolonged treatment (6-24 h) with HYS-32. Further, HYS-32 treatment induced GSK3β phosphorylation at Y216 and S9, where the ratio of GSK3β-pY216 to GSK3β-pS9 was first elevated followed by a decrease over time. Co-treatment of astrocytes with HYS-32 and GSK3β inhibitor SB415286 attenuated the HYS-32-induced microtubule catastrophes and partially prevented EB1 dissociation from the plus end of microtubules. Furthermore, co-treatment with PI3K inhibitor LY294002 inhibited HYS-32-induced GSK3β-pS9 and partially restored EB1 distribution from the microtubule lattice to plus ends. Together these findings suggest that HYS-32 induces microtubule catastrophes by preventing EB1 from targeting to microtubule plus ends through the GSK3β signaling pathway.

## Introduction

Astrocytes are the most abundant glial cell type that distribute throughout the central nervous system (CNS) and tile the entire CNS in a contiguous non-overlapping manner [1]. Astrocytes play an essential role in the CNS homeostasis and respond to multiple forms of CNS injury and diseases by changing their morphology, function, and gene expression [1]. The morphological alterations may be related to upregulation and reorganization of cytoskeletal proteins, including glial fibrillary acidic protein (GFAP), actin, and microtubule [2,3]. Microtubules are made of linear protofilaments which consist of α/β-tubulin heterodimers in a head-to-tail manner, thereby leading to the intrinsic polarity of microtubules [4]. Thirteen protofilaments associate laterally to organize as a sheet, which gradually closes to form the pseudo-helical structure for subsequent incorporation into the microtubule lattice [5]. Microtubules have two structurally different ends, the microtubule organizing center (MTOC)-anchored slow-growing minus ends, and the cell periphery-localized fast-growing plus ends [6]. Microtubule plus ends undergo random changes between two states of growth (polymerization) and shrinkage (depolymerization), and are separated by catastrophe (shift from growth to shrinkage state) or rescue (shift from shrinkage to growth state) events [7]. This highly dynamic behavior of microtubules named dynamic instability is powered by hydrolysis of guanosine triphosphate (GTP) [8]. Stabilization of microtubule plus ends at the cell cortex results in polarized microtubule arrangements and facilitates directional cell migration [9]. The intrinsic dynamic property of microtubules is dictated by microtubule-associated proteins (MAPs) that bind to the surface of microtubules or free tubulin subunits in the cytoplasm [10] via a phosphoinositide3-kinase (PI3K)-glycogen synthase kinase 3β (GSK3β) signaling mechanism [11,12]. Among the two major groups of MAPs, the conventional MAPs bind along the microtubule lattice, whereas microtubule plus end-tracking proteins (+TIPs) specifically localize to growing microtubule plus ends in a comet-like manner [13]. +TIPs control microtubule dynamic instability and attach microtubule tips to other intracellular structures, including centromeres and actin filaments [14]. Among the +TIPs, cytoplasmic linker protein 170 (CLIP-170), cytoplasmic linker associated proteins (CLASPs), adenomatous polyposis coli (APC), and end binding protein-1 (EB1) all play pivotal roles in cell migration by modulating microtubule dynamic instability [15]. EB1 preferentially associates with the growing microtubule plus ends to generate comet-like streaks (1–2 μm) on microtubule tips [16,17], facilitating cell migration and persistent microtubule growth [18]. It also acts as an adaptor protein to recruit other +TIPs at the plus ends of growing microtubules, thus EB1 is considered as the master regulator of microtubule dynamic instability [19]. HYS-32, a 4-(3,4-dimethoxyphenyl)-3-(naphthalen-2-yl)-2(5*H*)-furanone compound, is a novel derivative of a depolymerizing agent, combretastatinA-4 (CA-4) [20]. Our previous study has demonstrated that HYS-32 induces microtubule coiling in astrocytes [20]; however, the mechanism of how HYS-32 affects microtubule instability remained unknown. This study aims to elucidate the effect of HYS-32 on EB1-microtubule interaction and the signaling pathway involved in HYS-32-induced microtubule catastrophe in rat astrocytes.

## Materials and Methods

### Reagents and Antibodies

Compound HYS-32 was obtained from National Research Institute of Chinese Medicine (Dr. Chien-Chang Shen). Bovine Serum Albumin (A9647) was purchased from Sigma-Aldrich (St. Louis, MO). Fluorescence Mounting Medium (S3023) was purchased from Dako (Santa Clara, CA). Bio-Rad DC protein assay kit was purchased from Bio-Rad Laboratories (Hercules, CA). GSK3β inhibitor SB415286 and PI3K inhibitor LY294002 were purchased from Cayman Chemical (Ann Arbor, MI). Alexa Fluor 350 Phalloidin was purchased from Invitrogen (Carlsbad, CA). The primary antibodies used in this study were mouse monoclonal anti-β-tubulin (Sigma-Aldrich) and anti-GSK3β (pY216) (BD Biosciences, San Jose, CA) and rabbit polyclonal anti-human N-cadherin (TAKARA BIO INC., Otsu, Shiga, Japan), anti-β-tubulin (Santa Cruz, CA), anti-EB1 (Millipore, Temecula, CA), anti-human GSK3β (Cell Signaling, Danvers, MA), anti-GSK3α/β (S21/S9) (R&D Systems, Minneapolis, MN), and anti-glyceraldehyde-3-phosphate dehydrogenase (GAPDH; GeneTex, Irvine, CA). The secondary antibodies used were horseradish peroxidase (HRP)-conjugated goat anti-rabbit IgG (Chemicon, Temecula, CA), HRP-conjugated goat anti-mouse IgG (Promega, Madison, WI), Alexa Fluor 488-conjugated goat anti-mouse IgG (Life Technologies, Carlsbad, CA) and Alexa Fluor 594-conjugated goat anti-rabbit IgG (A11012; Life Technologies). Western Lightning Plus-ECL Oxidizing Reagent Plus, Enhanced Luminol Reagent Plus, and Protran nitrocellulose membranes were purchased from PerkinElmer Inc. (Waltham, MA). Protein G Mag Sepharose Xtra beads were purchased from GE Healthcare (Uppsala, Sweden). The 25 Culture-Inserts were purchased from ibidi (Munich, Germany).

### Ethics Statement

Animal protocols were approved by the Institutional Animal Care and Use Committee of National Yang-Ming University (IACUC permit number: 1021220). Humane care for all animals was observed, in compliance with the Guide for the Care and Use of Laboratory Animals as adopted and promulgated by the United States National Institutes of Health (NIH publication No. 85–23, revised 1985).

### Neonatal Rat Astrocyte Primary Culture

Two-day-old Sprague-Dawley rats of both sexes were provided by the Laboratory Animal Center of the National Yang-Ming University. Primary astrocytes were dissociated from cerebral cortex of neonatal rats as previously described [21].

### Drug Treatments

HYS-32 was dissolved in dimethyl sulfoxide (DMSO) to obtain a stock solution (5 mM). Primary astrocytes at 90% confluence were treated with 5 μM of HYS-32 at 37°C for the indicated time. In GSK3β inhibition experiments, the astrocytes were treated for 24 h with 5 μM of HYS-32 alone or co-treated with the GSK3βinhibitor SB415286 (20 μM). In PI3K inhibition experiments, the astrocytes were treated for 24 h with 5 μM of HYS-32 alone or co-treated with the PI3K inhibitor LY294002 (20 μM). Control astrocytes were incubated with 0.2% DMSO alone. InHYS-32 removal experiments, the astrocytes were treated for various time periods with 5 μM of HYS-32, were then removed from the HYS-32 and rinsed twice with growth medium to recover for 1–24 h. The astrocytes were processed for immunofluorescence and confocal microscopy, immunoblot analysis, and immunoprecipitation.

### Immunofluorescence Confocal Microscopy and Image Analysis

Primary astrocytes were processed for immunofluorescence as described previously with minor modifications [20]. Astrocytes plated on collagen-coated glass coverslips were fixed with acetone for 10 min at -20°C, and rinsed twice with PBS. The cells were incubated at 4°C for 16–18 h with a mixture of mouse antibody against β-tubulin (1:200) and rabbit antibody against N-cadherin (1:200), or rabbit antibody against EB1 (1:200) in PBS. After washing with PBS three times for 5 min, the cells were incubated with a mixture of Alexa Fluor 488 goat anti-mouse IgG antibody and Alexa Fluor 594 goat anti-rabbit IgG antibody (both 1:150) in the dark for 1 h at room temperature. After washing with PBS three times for 5 min, the cells were incubated with Alexa Fluor 350 Phalloidin (1:20) in the dark for 1 h at room temperature. Subsequently, the cells were rinsed with PBS then mounted in Fluorescence Mounting Medium and sealed with nail polish. The labeled cells were examined using a Leica Microsystems microscope (Leica, Wetzlar, Germany) equipped for epifluorescence. Images were acquired using a 100×oil-immersed objective and digitized using a BD Spinning-discConfocal and Real-time Vision (CARV) II (BD Bioscience). No labeling was visible when both primary antibodies were omitted, nor fluorescence signal bleed-through between filters was detected when only one primary antibody was omitted in double labeled samples. Quantitative analysis of the straight distance between microtubule tips and the cell border were performed for at least 120 microtubules on immunofluorescence images upon staining of astrocytes for β-tubulin and F-actin, or N-cadherin by using Image-Pro Plus software in three independent experiments. F-actin and N-cadherin staining were used to mark the cell border.

### Immunoblot Analysis

Primary astrocytes grown on the rat tail collagen-coated 35 mm or 60 mm culture dishes were washed with ice cold PBS, then lysed and harvested on ice in 100 μl modified RIPA buffer (50 mM Tris-HCl, pH 7.4, 1% nonidet-P40, 150 mM NaCl, 1 mM EDTA), containing protease inhibitors (1 mM PMSF and 1 μg/ml each of pepstatin, leupeptin, and aprotinin) and phosphatase inhibitors (1 mM NaF and 1 mM Na_3_VO_4_). Afterward, cells were sonicated for 5–10 sec on ice. The concentration of protein samples were measured by the Lowry method using Bio-Rad DC protein assay kit at 750 nm in a spectrophotometer (DU800; BeckmanCoulter, Brea, CA). The protein samples were blended gently with reducing Laemmli sample buffer (40% glycerol, 20% β-mercaptoethonal, 8% SDS, 0.012% bromophenol blue, and 0.25 M Tris-HCl; pH 6.8), boiled for 5 min, and then stored at -20°C. Protein samples (20μg per lane) were separated by electrophoresis on 10% SDS-PAGE then transferred to Protran nitrocellulose membranes (Whatman, PerkinElmer). Strips of the membranes were blocked at room temperature for 1 h in blocking buffer [5% non-fat milk or 2.5% BSA in TBST (150 mM NaCl, 0.1% Tween-20, and 50 mM Tris-HCl; pH 7.4)]. Subsequently, they were incubated overnight at 4°C with mouse monoclonal against β-tubulin (1:2000) or mouse polyclonal against GSK3β (pY216) (1:3000) or rabbit polyclonal against EB-1 (1:3000), against GAPDH (1:5000), or against GSK3α/β (S21/S9) (1:2000). After washing with TBST three times for 5 min (150 mM NaCl, 0.1% Tween-20, and 50 mM Tris-HCl; pH 7.4), the blots were incubated with HRP-conjugated goat anti-mouse IgG (1:3000) or anti-rabbit IgG (1:20,000) for 1 h at room temperature, followed by washing three times with TBST (pH 7.4). Immunoreactive bands were developed by ECL reagents (PerkinElmer Inc.) and exposed to X-ray film (FUJIFILM, Japan). The photographic bands on the films were scanned by EPSON PERFECTION V10 and quantified by densitometric analyses using Gel-Pro Analyzer 3.1 software.

### Immunoprecipitation

Primary astrocytes were manipulated for immunoprecipitation as previously reported with minor alterations [22]. Whole cell lysates were lysed and harvested on ice in 200 μl of modified RIPA buffer (50 mM Tris-HCl, 1% Noidet-P40, 0.5% Triton X-100, 150 mM NaCl, 1 mM EDTA, pH 7.4), containing protease inhibitors (1 mM PMSF and 1 μg/ml each of pepstatin, leupeptin, and aprotinin) and phosphatase inhibitors (1 mM NaF and 1 mM Na_3_VO_4_). After being incubated on ice for 10 min, lysates were removed by centrifugation at 12,300×g for 10 min at 4°C, then pre-cleared with 20 μl Protein G Mag Sepharose Xtra beads (GE Healthcare). The prepared sample lysates were added to the Protein G Mag Sepharose Xtra beads (50 μl) which were pre-coated with rabbit or mouse polyclonal antibodies against β-tubulin (0.5 μg) in each microcentrifuge tube and incubated at 4°C overnight on a rotating apparatus with an extra 1 mM PMSF. The Mag Sepharose-bound immunoreactive complexes were washed 6 times with 500 μl modified RIPA buffer. The pellets were re-suspended in reducing Laemmli sample buffer (40% glycerol, 20% β-mercaptoethonal, 8% SDS, 0.012% bromophenol blue, and 0.25 M Tris-HCl; pH 6.8) to each tube. Denatured proteins were released from the Mag Sepharose beads by heating at 95°C for 5 min, then the supernatant was collected into microcentrifuge tubes and stored at -20°C. Immunoblot analyses were performed according to the above procedures.

### Migration Assay

Migration assay was performed using the ibidi Culture-Insert (ibidi) based in part on a previously reported method [23]. This instrument provides two wells with a separation wall of 500 μm thick. The culture-inserts were placed in the individual wells of a 24-well plate, then 70 μl of astrocyte suspension (4×10^5^ cells/ml) was seeded into each well of the insert. The cell migration into the cell free area was observed under a Nikon TS 100 inverted microscope (Nikon, Tokyo, Japan). Images were acquired with a Canon EOS 450D digital camera (Canon, Tokyo, Japan). Measurement of astrocytes migration area was performed using Image-Pro Plus software.

### Statistical Analysis

All data are expressed as the mean ± standard deviation (SD). All experiments were performed at least three times and analysis of the results was assessed by one-way ANOVA test followed by Dunnett’s post hoc tests using SPSS 12.0 software. A*P* value < 0.01 was considered significant.

## Results

### HYS-32 Induces Microtubule Catastrophes and Prevents Microtubules Targeting to Cell Cortex in a Dose- and Time-Dependent Manner

**Fig 1:**
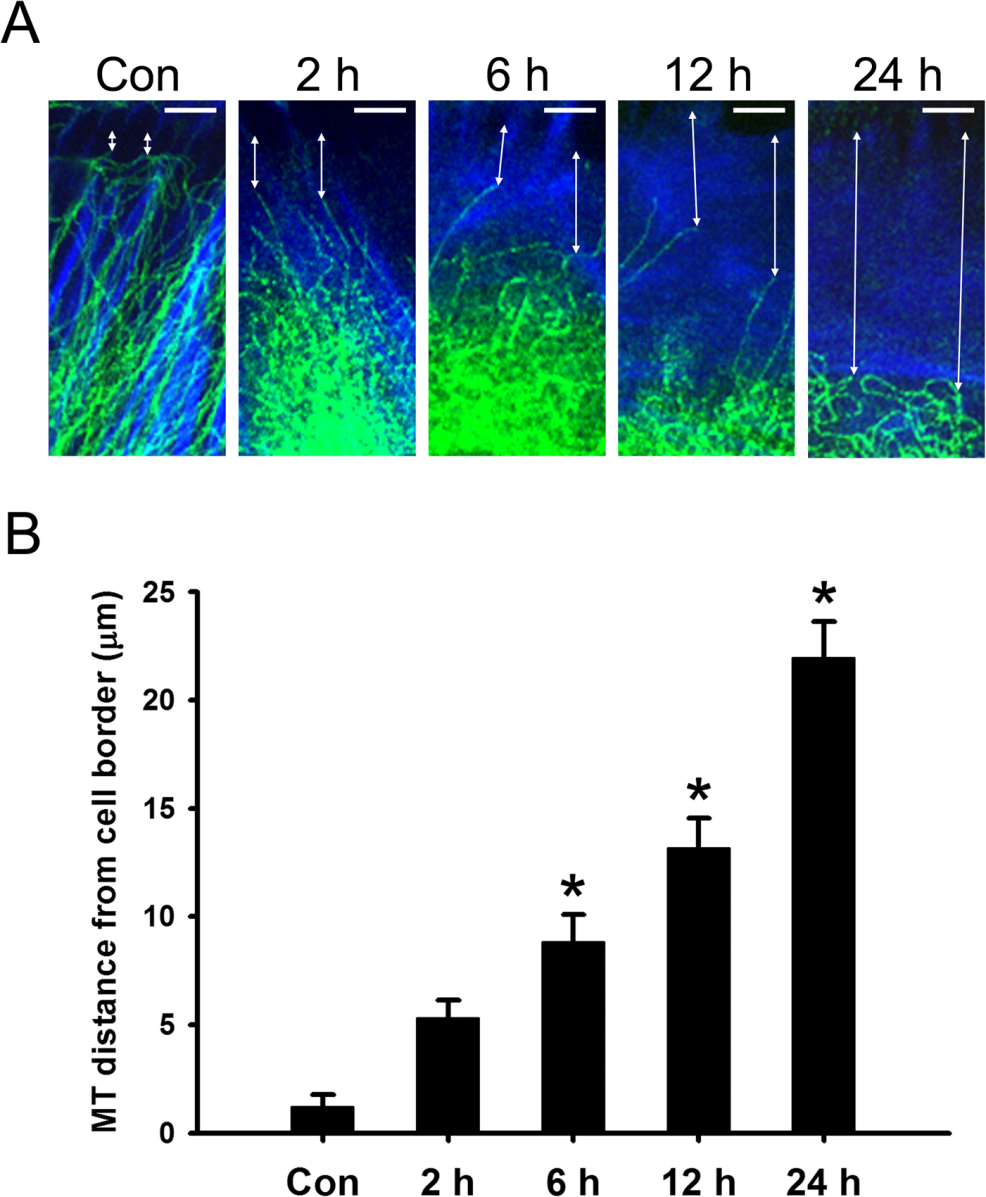
HYS-32 prevents microtubules from targeting to cell cortex. (A) Control astrocytes (Con) or astrocytes treated for 2, 6, 12, or 24 h with 5 μM HYS-32 were fixed in cold acetone and double-stained for β -tubulin (green) and F-actin (blue). Double arrows indicate the distance between microtubule tips and the cell border (bars = 5 μm). (B) Quantitative analyses of the distance between microtubule tips and cell border were performed as described in Materials and Methods. The results were collected from three independent experiments. *p<0.01compared to control (Con) using one-way ANOVA with Dunnett’s post-hoctest.

To investigate the dose-dependent effect of HYS-32 on microtubule in astrocytes, cells were treated for 24 h with various concentrations (0.5, 1, 2, 5, and 10 μM) of HYS-32. Confocal microscopy with double immunofluorescence staining of β-tubulin and N-cadherin showed that bundles of microtubules radiated out the surrounding area of the nucleus and extended toward the cell periphery in control astrocytes (S1 Fig, Con). At concentrations higher than 1 μM, HYS-32induced a disorderly coiled pattern on microtubules (S1 Fig). Higher concentration of HYS-32 (5 μM) induced retraction of microtubules from the cell border and coiled up at perinuclear regions. Treatment of astrocytes with 10 μM HYS-32 resulted in a partial disassembly of the microtubule (S1 Fig). The concentration of 5 μM HYS-32 was selected for use in all following experiments in this study. In the time course experiment, short-term (0.5–1 h) HYS-32 treatment had little effect on microtubule morphology in astrocytes (S2 Fig); however, longer exposure (≥1.5 h) of HYS-32 induced microtubule retraction and coiling at the surrounding area of nucleus. This phenomenon persisted for at least 24 h (S2 Fig). Furthermore, HYS-32 treatment had no effect on GFAP expression (data not shown) or F-actin distribution (S3 Fig), we thus used F-actin staining to mark the cell border, which was applied for measuring the distance between cell periphery and microtubule tips. In order to confirm the detailed effect of HYS-32 on microtubule retraction from the cell border, astrocytes were double-labeled for β-tubulin and F-actin, and the distances between microtubule tips and the cell border were measured and quantified. As time passed on, it became more obvious that microtubules retracted from the cell border and coiled up (Fig 1A). In control astrocytes, the average distance between microtubule tips and the cell border was 1.22 μm; however, the distance greatly increased from 5.31 μm (2 h) up to 21.95 μm (24 h) in HYS-32-treated astrocytes (Fig 1B).

### HYS-32 Eliminates EB1 Staining From the MT Plus Ends and Induces an Extensive Distribution of EB1 on the Microtubule Lattice

**Fig 2:**
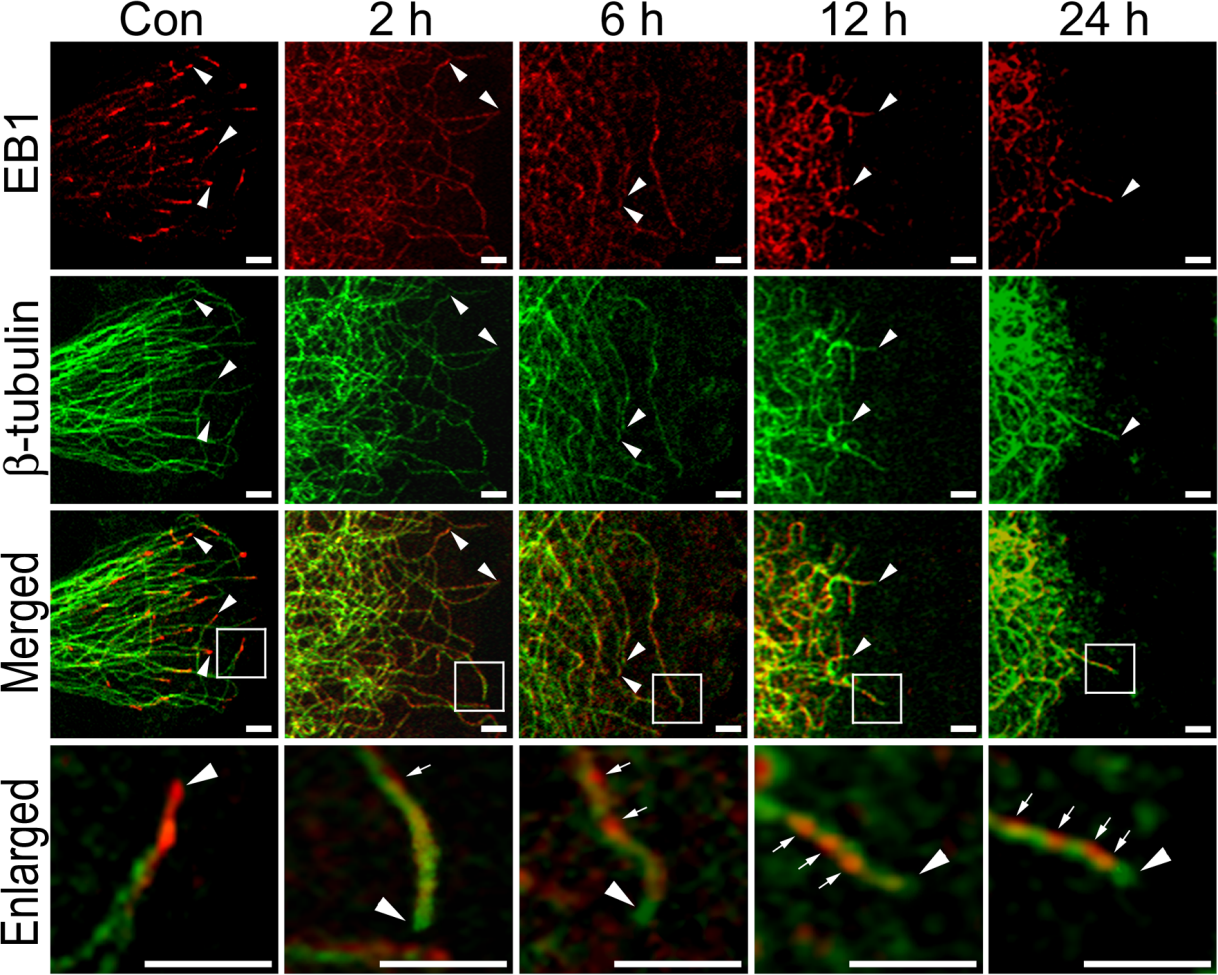
HYS-32 induces extensive distribution of EB1 on the microtubule lattice and eliminates EB1 staining from the microtubule plus end. Control astrocytes (Con) or astrocytes treated for 2, 6, 12, or 24 h with 5 μM HYS-32 were fixed in cold acetone and double-stained for β-tubulin (green) and EB1 (red) and subjected to confocal microscopy. Images were merged to show co-localization (Merged). Square areas were enlarged to show EB1 distribution (Enlarged). Arrowheads indicate microtubule tips. Arrows indicate distribution of EB1 along the microtubules (bars = 5 μm).

Confocal microscopy of control astrocytes with double immunofluorescence staining of β-tubulin and EB-1 showed that EB1 concentrated at growing microtubule plus ends as typical bright comet-like streaks (Fig 2, Con, arrowheads). HYS-32 treatment for 2 h depleted EB1 comet-like streaks from microtubule plus ends, and this depletion sustained for up to 24 h (Fig 2, Enlarged, arrowheads). HYS-32 treatment also caused accumulation of EB1 on microtubule lattice (Fig 2, Enlarged, arrows) and EB1 accumulation on microtubule lattice became more apparent after prolonged HYS-32 treatment (Fig 2, 6–24 h). By 24 h, most of the microtubules were bunched up as a coiled mass in the perinuclear area and only few tips remained at the cell cortex (S4 Fig, arrowheads), where the EB1 signal has apparently moved away from the tips (Fig 2, 24 h, arrowheads). Although HYS-32 caused a dramatic alteration of EB1 distribution on microtubule network and the EB1 expression appeared to be increased following HYS-32 exposure (Fig 2), levels of EB1 and β-tubulin proteins were unchanged as demonstrated by immunoblot analysis (S5 Fig).

### Removal of HYS-32 Restores EB1 Comet-like Streaks and Microtubule Distribution to Cell Cortex

**Fig 3:**
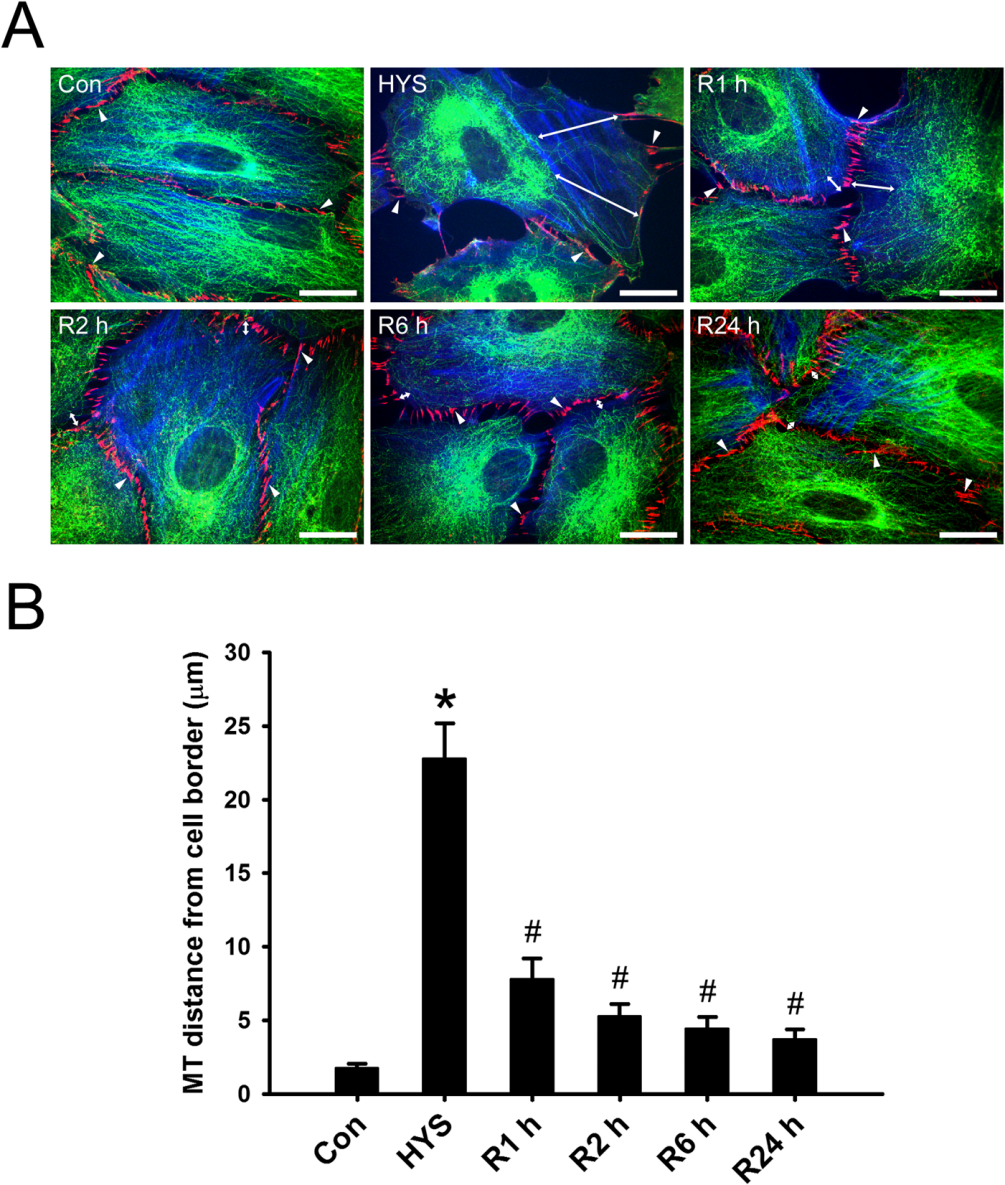
Removal of HYS-32 rescues microtubule catastrophes. (A) Control astrocytes (Con), astrocytes treated for 24 h with 5 μM HYS-32 (HYS), or astrocytes treated for 24 h with HYS-32 then replaced with normal culture medium for 1 to 24 h (R1 h, R2 h, R6 h, or R24 h) in the absence of HYS-32 were fixed in cold acetone and triple-stained for β-tubulin (green), N-cadherin (N-cad), and F-actin (blue). Arrowheads indicate the intercellular junctions. Double-arrows indicate the distance between microtubule tips and cell border (bars = 20 μm). (B) Quantification of the mean distances between microtubule tips and cell border. The results were collected from three independent experiments. *p<0.01 compared to control, ^#^ *p*<0.01 compared to HYS using one-way ANOVA with Dunnett’s post-hoc test.

**Fig 4:**
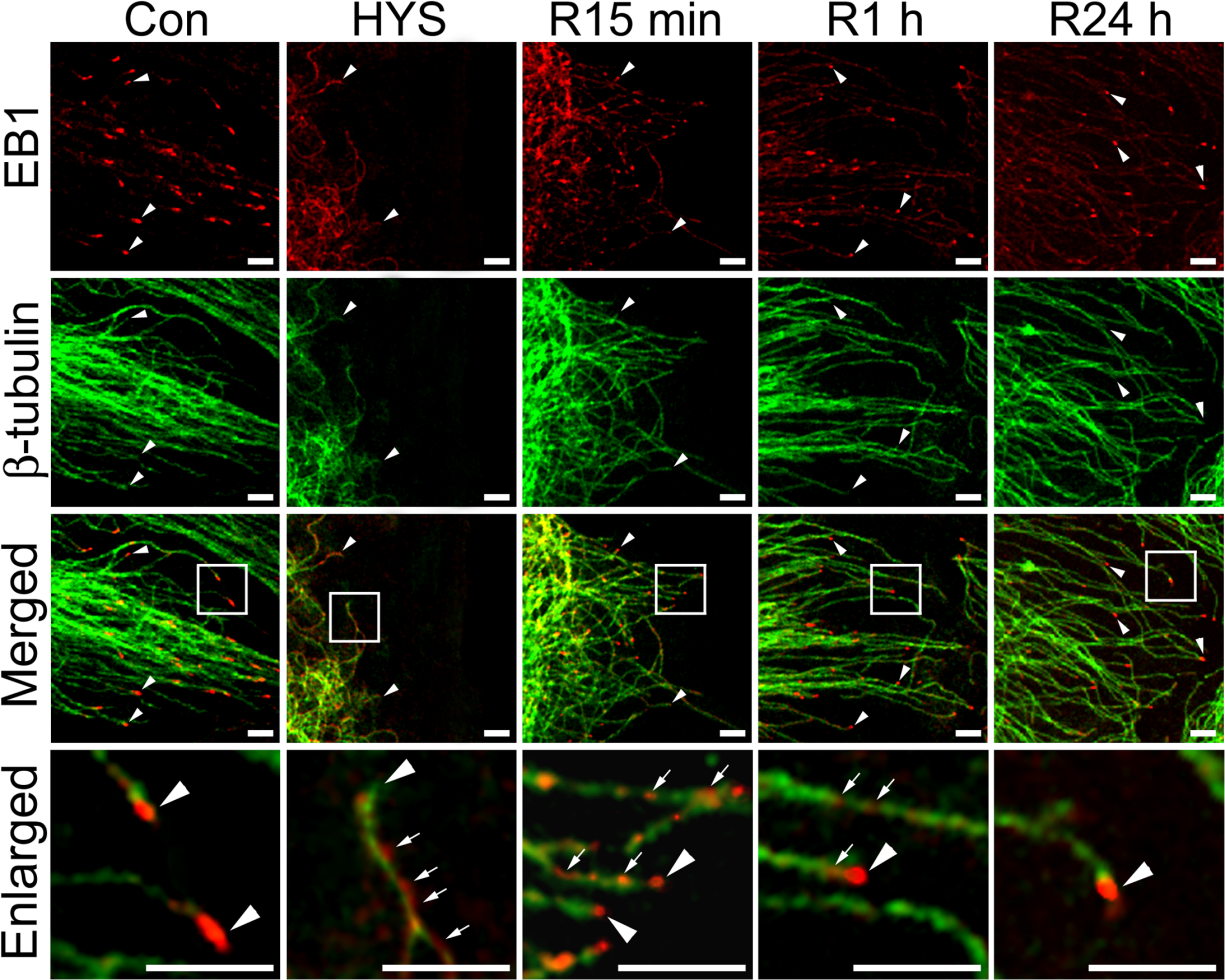
Removal of HYS-32 restores EB1 comet-like streaks. Control astrocytes (Con) or astrocytes treated for 24 h with 5 μM HYS-32 (HYS), or treated for 24 h with 5 μM HYS-32 then replaced with normal culture medium for 15 min to 24 h (R15 min, R1 h, or R24 h) were fixed in cold acetone and double-stained for β-tubulin (green) and EB1 (red), and subjected to confocal microscopy. Images were merged to show co-localization (Merged). Square areas were enlarged to show EB1 distribution (Enlarged). Arrowheads indicate microtubule tips. Arrows indicate distribution of EB1 along the microtubules (bars = 5μm).

We next examined whether the HYS-32-induced effect on microtubule is reversible by placing HYS-32-treated astrocytes into a normal culture medium. As shown in Fig 3, the distance between microtubule tips and the cell border was 1.79 μm in control astrocytes, and greatly increased to 22.83 μm in astrocytes treated for 24 h with HYS-32 (Fig 3B); however, the distances between microtubule tips and the cell border were significantly reduced when HYS-treated astrocytes were switched to a normal culture medium for various time periods (1, 2, 6, and 24 h), and no difference was observed at 24 h as compared to control astrocytes (Fig 3B). Placement of the HYS-treated astrocytes into normal culture medium for 15 min restored the distribution ofEB1 comet-like streaks at microtubule plus ends accompanied by an extension of retracted microtubules to the cell cortex (Fig 4, R15 min). Longer exposure with a normal culture medium showed a more prominent distribution of EB1 streaks at microtubule plus ends (Fig 4, R1 h, R24 h). Diminished EB1 accumulation on microtubule lattice was also noted at 1 h (Fig 4, R1 h), and only weak EB1 staining was found on microtubule lattice at 24 h following medium replacement (Fig 4, R24 h).

### HYS-32 Affects the Association between EB1 and β-tubulin

**Fig 5:**
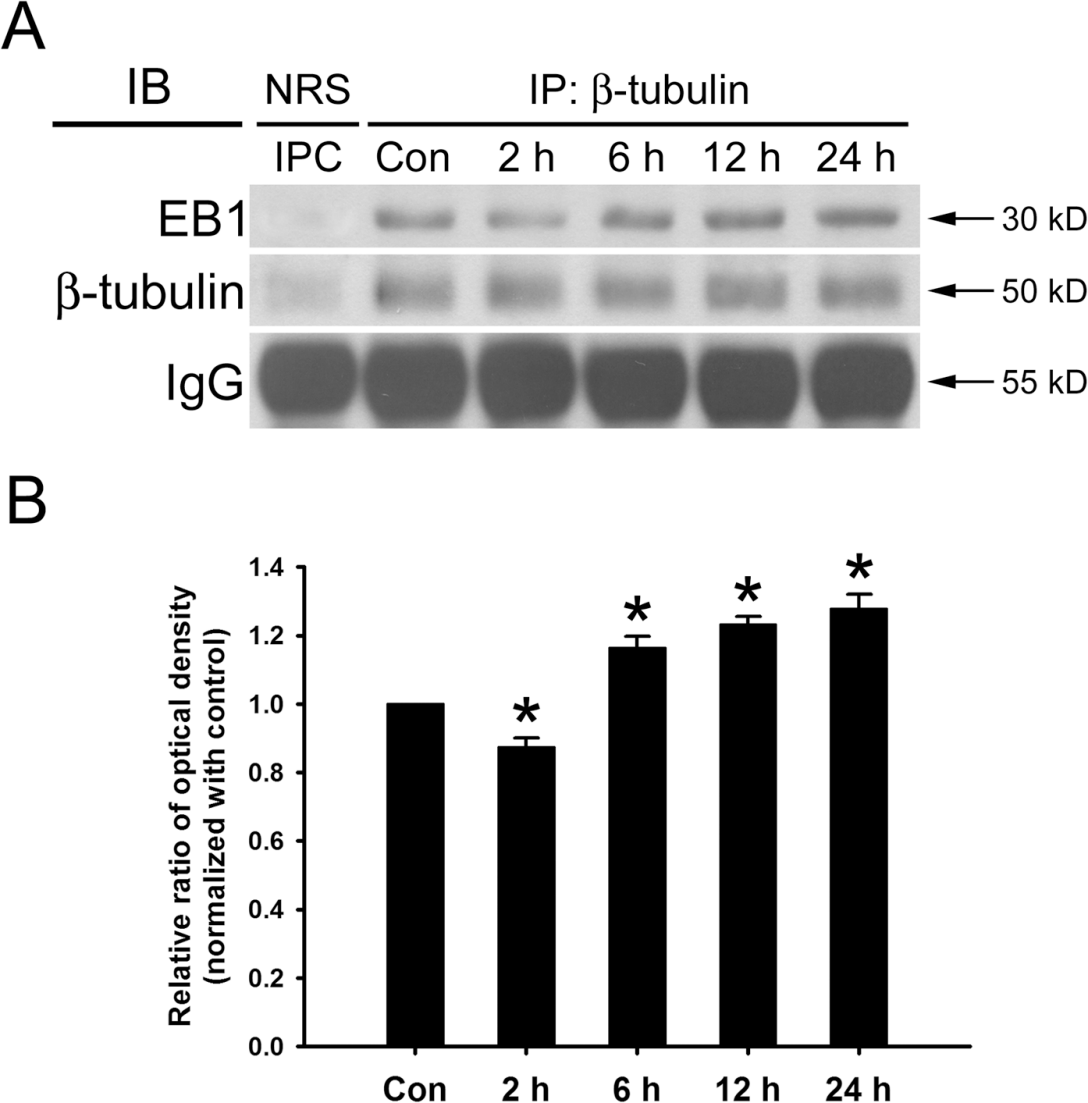
HYS-32 affects the association between EB1 and β-tubulin. (A) Cell lysates from control astrocytes (Con) or astrocytes treated for 2, 6, 12, or 24 h with 5 μM HYS-32. The cell lysates were immunoprecipitated using normal rabbit serum (NRS) as immunoprecipitation control (IPC) or mouse antibody against β-tubulin (IP: β-tubulin). The immunoprecipitates were then subjected to 10% SDS-PAGE, and analyzed by immunoblotting with antibodies against -tubulin (IB: -tubulin) or EB1 (IB: EB1). (B) Densitometric analyses of EB1 expressed as the density of the bands in the treated groups relative to the control. The results were collected from three independent experiments.*p<0.01 compared to control using one-way ANOVA with Dunnett’s post-hoc test.

Since HYS-32 caused a notable alteration of EB-1 distribution on microtubule, we further inspected whether the association between EB-1 and β-tubulin was influenced by HYS-32. Immunoprecipitation of HYS-32 treated cells at different time points were carried out using polyclonal antibody against β-tubulin, followed by immunoblotting of the immunoprecipitated proteins with a polyclonal anti-EB1 antibody. As displayed in Fig 5, HYS-32 treatment for 2 h caused a slight decrease in the association between EB-1 and β-tubulin (Fig 5A, 3^rd^ lane; 5B); however, exposure of HYS-32 for longer time periods from 6–24 h caused increased association between EB-1 and β-tubulin (Fig 5A, 4^th^, 5^th^, 6^th^ lanes, 5B).

### HYS-32 Modulates GSK3β Signaling Pathway

**Fig 6:**
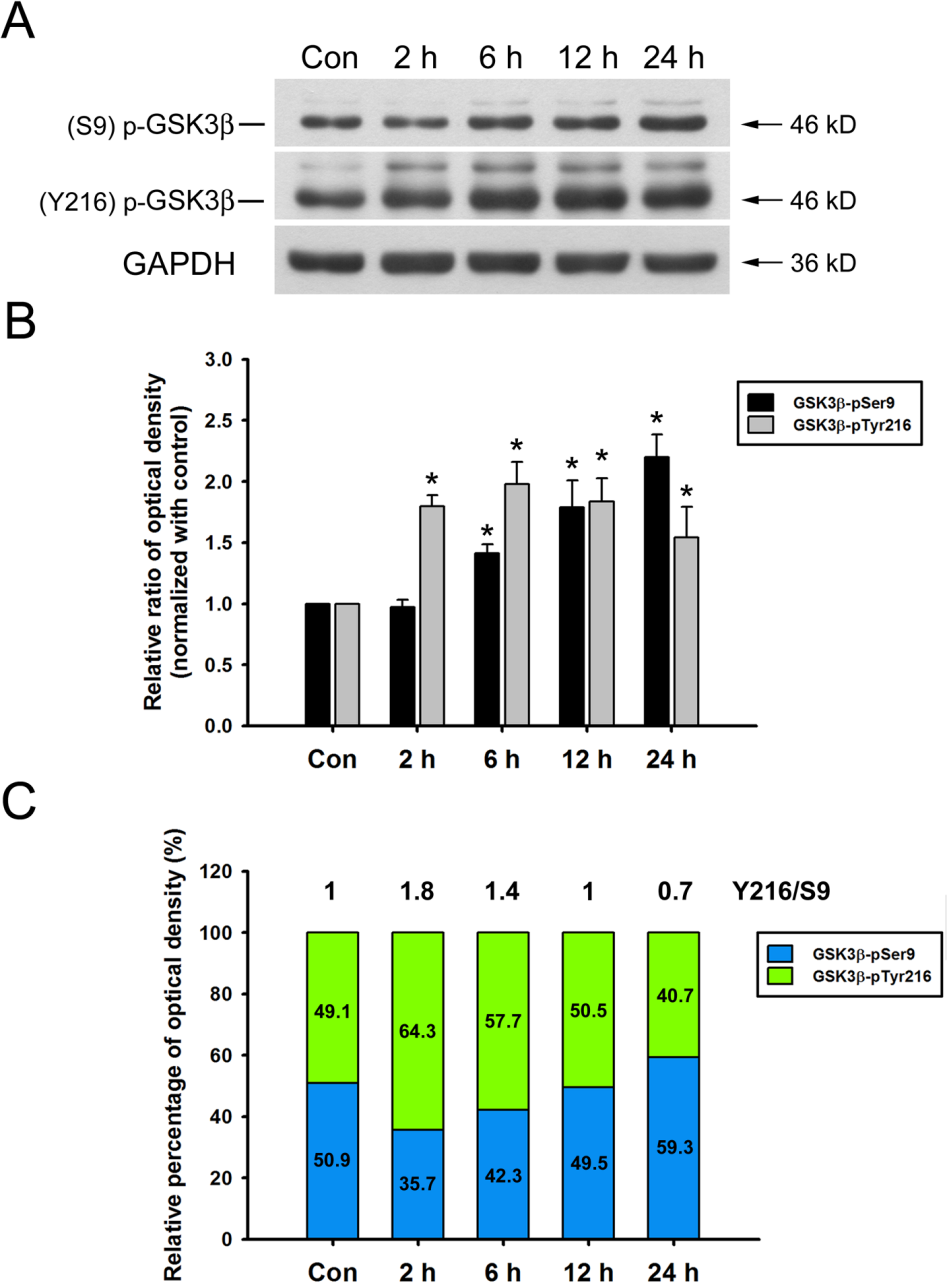
HYS-32 modulates GSK3β phosphorylation. (A) Cell lysates from control astrocytes (Con) or astrocytes treated for 2, 6, 12, or 24 h with 5 μM HYS-32 were subjected to 10% SDS-PAGE, and analyzed by immunoblotting with antibodies against GSK3β-pTyr216, GSK3β-pSer9, or GAPDH. (B) Densitometric analyses of GSK3β-pTyr216 and GSK3β-pSer9 expressed as the density of the bands in the treated groups relative to the control. *p<0.05 compared to control using one-way ANOVA with Dunnett’s post-hoc test. The results were collected from five independent experiments. (C) According to the data in (B), the stacked bar graph showing the relative percentage of phospho-GSK3β at indicated time.

We next explored the signaling pathway which participates in HYS-induced microtubule catastrophes. From time-course experiments, the levels of GSK3β phosphorylated at tyrosine 216 residue (GSK3β-pY216) rapidly increased at 2 h following HYS-32 treatment, reached its peak at 6 h, and followed by a gradual decline at 24 h (Fig 6A and 6B). Levels of GSK3β phosphorylated at serine 9 residue (GSK3β-pS9) gradually increased after 6–12 h and peaked at 24 h (Fig 6A and 6B). Quantitative analyses revealed that the ratio of GSK3β-pY216 to GSK3β-pS9 (Y216/S9) was about 1 in control astrocytes (Fig 6C, Con), and rapidly increased to 1.8 during the initial 2 h, and gradually declined after 6, 12, and 24 h (1.4, 1, and 0.7, respectively) of HYS-32 treatments (Fig 6C, 2, 6, 12, and 24 h). These observations indicated that HYS-32 induced an apparent shift of GSK3β phosphorylation state over time.

### GSK3β Inhibitor SB415286 Attenuates HYS-32-induced Microtubule Catastrophe and Partially Restores EB1 Distribution on Microtubule Plus Ends

**Fig 7:**
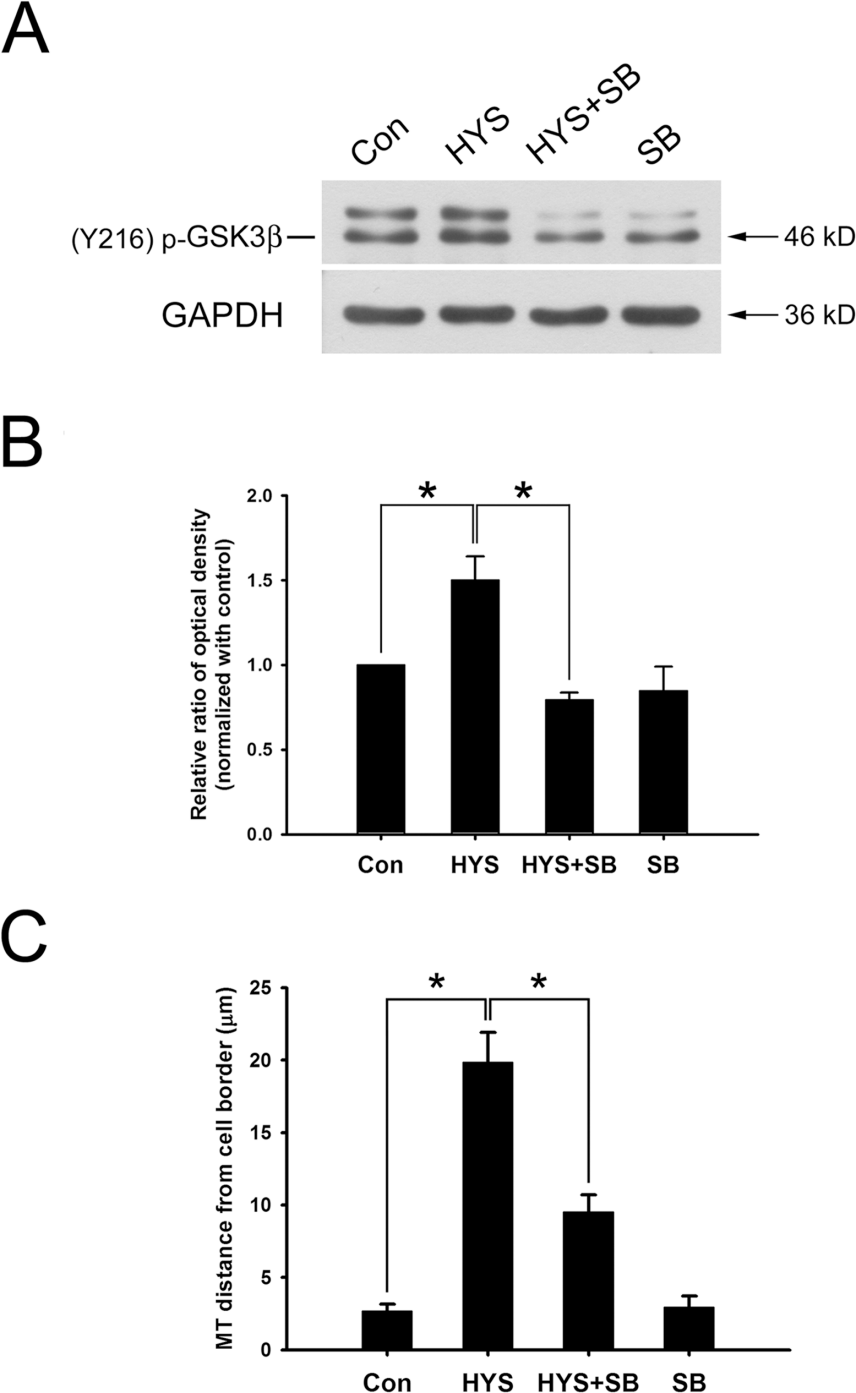
GSK3β inhibitor SB415286 inhibits the HYS-32-induced phosphorylation of GSK3β-pY216 and attenuates the HYS-32-induced microtubule catastrophes. (A) Control astrocytes (Con) or astrocytes treated for 24 h with 5 μM HYS-32 (HYS), co-treated for 24 h with 5μM HYS-32and 20μM SB415286 (HYS+SB), or treated with 20 μM SB415286 (SB) were subjected to 10% SDS-PAGE, and analyzed by immunoblotting with antibodies against GSK3β-pTyr216 or GAPDH. (B) Densitometric analyses of GSK3β-pTyr216 expressed as the density of the bands in the treated groups relative to the control. (C) Astrocytes treated as in (A) were fixed in cold acetone and triple-stained for β-tubulin, N-cadherin, and F-actin. Quantitative analysis of the straight distance between microtubule tips and cell border were performed as described in Materials and Methods. The results were collected from three independent experiments. *p<0.01 compared to HYS using one-way ANOVA with Dunnett’s post-hoc test.

**Fig 8:**
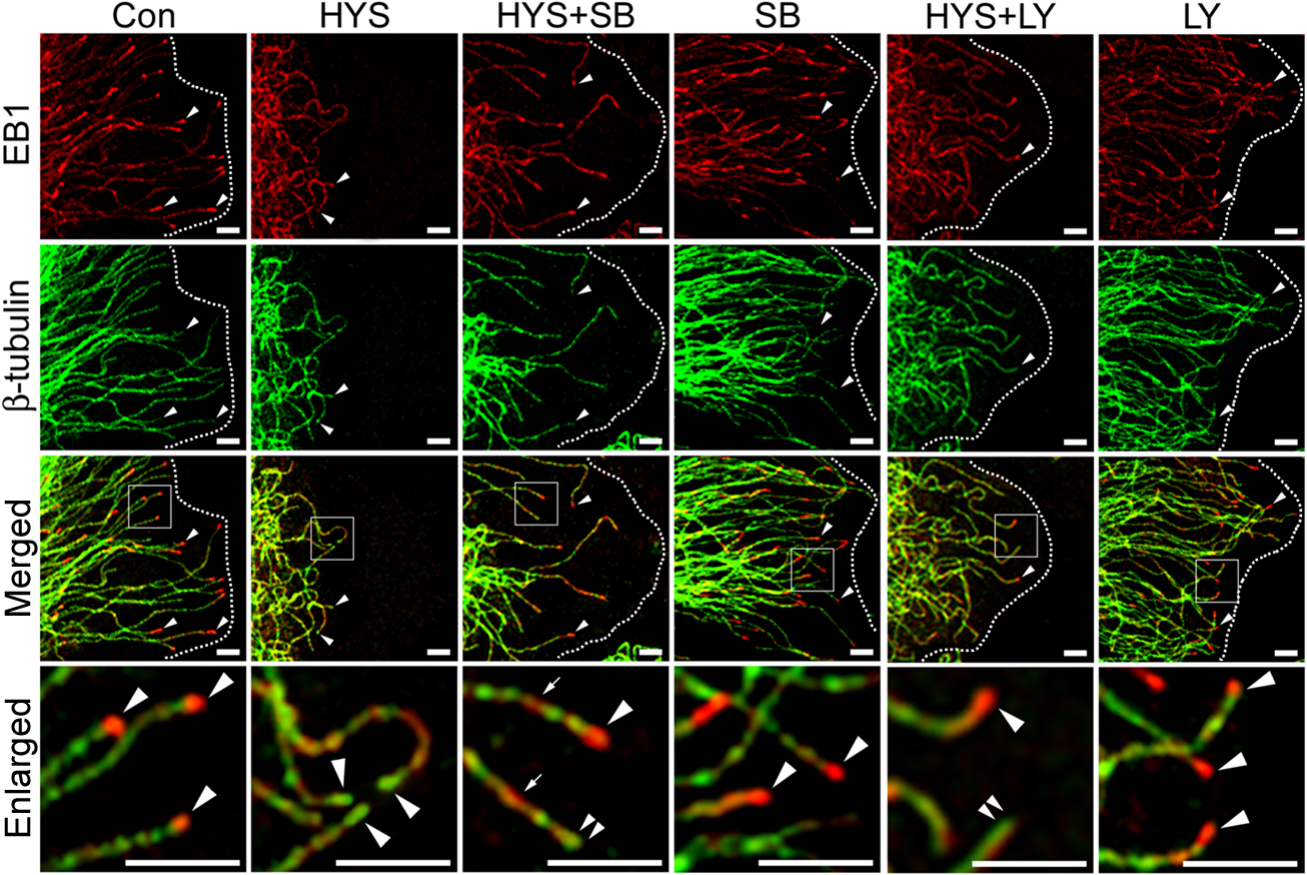
SB415286 and LY294002 partially restore EB1 distribution on microtubule plus ends. Control astrocytes (Con) or astrocytes treated for 24 h with 5 μM HYS-32 (HYS), co-treated for 24 h with 5 μM HYS-32 and 20μM SB415286 (HYS+SB) or LY294002 (HYS+LY), or treated with 20 μM SB415286 (SB)or LY294002 (LY) were fixed in cold acetone and double-stained for β-tubulin (green) and EB1 (red) and subjected to confocal microscopy. Images were merged to show co-localization (Merged). Square areas were enlarged to show EB1 distribution (Enlarged). Arrowheads indicate microtubule tips. Double-arrowheads indicate microtubule tips without EB1 signals. Arrows indicate distribution of EB1 along the microtubules. Dashed lines mark the cell border (bars = 5μm).

We next examined whether the effects of HYS-32 on microtubule catastrophes and EB1 distribution in astrocytes were mediated through the GSK3β-pY216 signaling pathway. As shown by the immunoblot analysis in Fig 7, treatment of astrocytes with HYS-32 for 24 h (Fig 7A and 7B, HYS) caused a notable increase in GSK3β-pY216 levels when compared to control astrocytes (Fig 7A and 7B, Con). The HYS-32-induced increase in GSK3β-pY216 protein levels were inhibited by co-treatment with GSK3β inhibitor SB415286 in HYS-32-treated astrocytes (Fig 7A and 7B, HYS+SB). Quantitative analyses showed that HYS-32 treatment caused a significant increase in distance between microtubule tips and the cell border (Fig 7C, HYS) as compared to the controls (Fig 7C, Con). The HYS-32-increased microtubule tips and cell border distances were greatly decreased by co-treatment with SB415286 in the HYS-32-treated astrocytes (Fig 7C, HYS+SB); however, confocal microscopy revealed that the HYS-32-induced depletion of EB1 comet-like streaks (Fig 8, HYS, arrowheads) on microtubule plus ends were only partially recovered by co-treatment of SB415286 in HYS-32-treated astrocytes (Fig 8, HYS+SB; compare arrowheads and double arrowheads in Enlarged). Treatment of SB415286 alone had no effects on microtubule tips and the cell border distance (Fig 7C, SB) and EB1 distribution (Fig 8, SB) in astrocytes.

### PI3K Inhibitor LY294002 Inhibits HYS-32-induced Phosphorylation of GSK3β-pS9 and GSK3β-pY216 and Partially Restores EB1 Distribution on Microtubule Plus Ends

**Fig 9:**
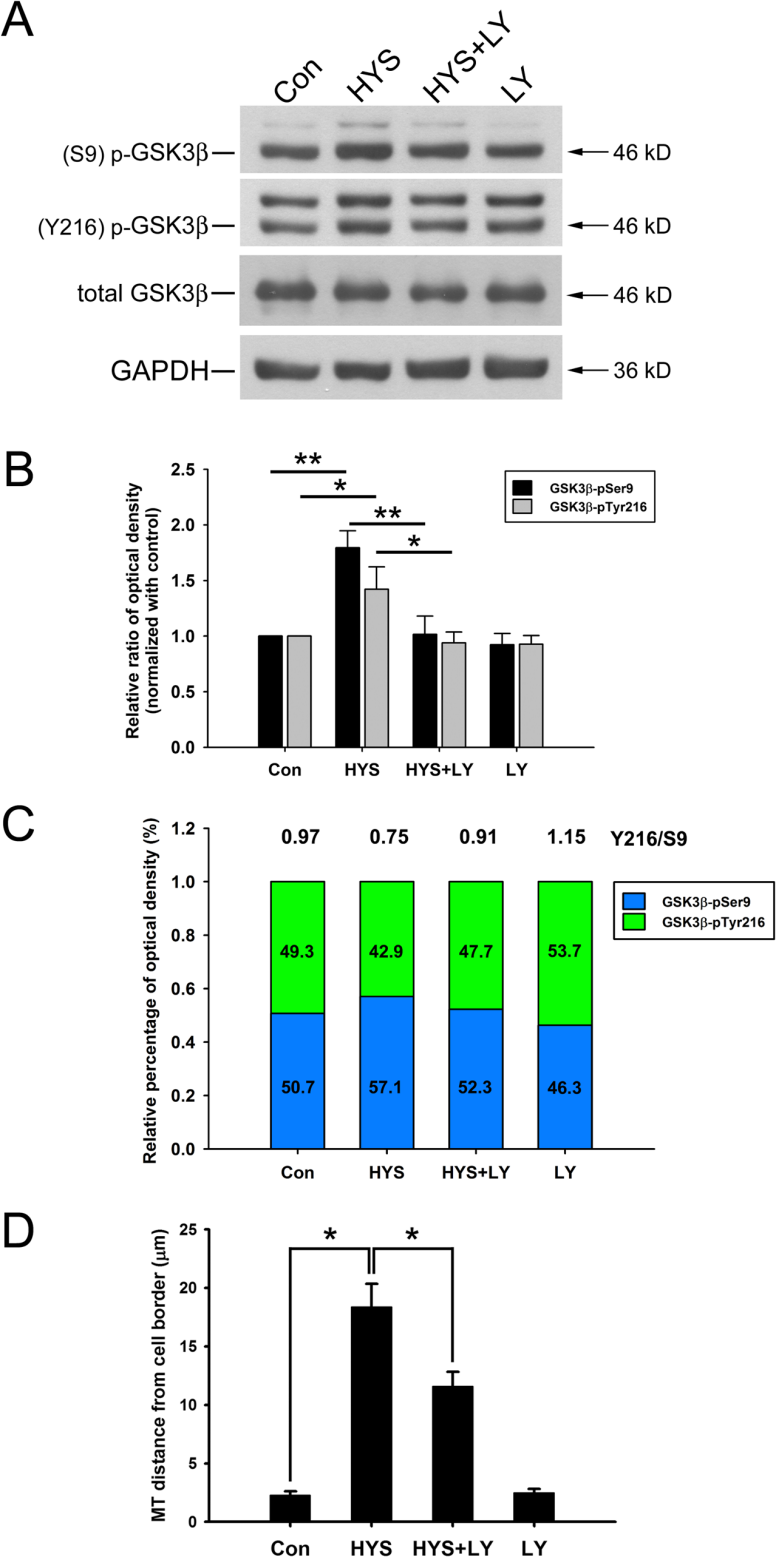
LY294002 inhibits the HYS-32-induced phosphorylation of GSK3β-pS9 and GSK3β-pY216. (A) Control astrocytes (Con) or astrocytes treated for 24 h with 5 μM HYS-32 (HYS), co-treated for 24 h with 5 μM HYS-32 and 20 μM LY294002 (HYS+LY), or treated with 20 μM LY294002 (LY) were subjected to 10% SDS-PAGE, and analyzed by immunoblotting with antibodies against GSK3β-pSer9, GSK3β-pY216, totalGSK3β, or GAPDH. (B) Densitometric analyses of GSK3β-pSer9 and GSK3β-pY216 expressed as the density of the bands in the treated groups relative to the control. (C) According to the data in (B), the stacked bar graph showing relative percentage ofphospho-GSK3β in various treatment. (D)Astrocytes treated as in (A) were fixed in cold acetone and triple-stained for β-tubulin, N-cadherin, and F-actin. Quantitative analysis of the straight distance between microtubule tips and cell border were performed as described in Materials and Methods. The results were collected from three independent experiments.*p<0.01 compared to HYS using one-way ANOVA with Dunnett’s post-hoctest.

We next examined whether the effects of HYS-32 on EB1 distribution in astrocytes were mediated through the GSK3β-pS9 signaling pathway by inhibiting PI3K. As shown in Fig 9, treatment of astrocytes with HYS-32 for 24 h (Fig 9A and 9B, HYS) caused a significant increase in both GSK3β-pS9 and GSK3β-pY216 protein levels as compared to the control astrocytes (Fig 9A and 9B, Con). The HYS-32-induced elevation in GSK3β-pS9 and GSK3β-pY216 levels was both inhibited by co-treatment with PI3K inhibitor LY294002 in HYS-32-treated astrocytes (Fig 9A and 9B, HYS+LY). Ratio of GSK3β-pY216 to GSK3β-pS9 (Y216/S9) was about 0.97 in control astrocytes (Fig 9C, Con), declined to 0.75 after 24 h of HYS-32 treatments (Fig 9C, HYS), and back to 0.91 by co-treatment with HYS-32 and LY294002 (Fig 9C, HYS+LY). Levels of total GSK3β protein were unchanged in LY294002-treated astrocytes as demonstrated by immunoblot analysis (Fig 9A). HYS-32 treatment caused a significant increase in distance between microtubule tips and cell border (Fig 9D, HYS) as compared to the controls (Fig 9D, Con). The microtubule tips and the cell border distances increased by HYS-32 were significantly decreased by co-treatment with LY294002 in the HYS-32-treated astrocytes (Fig 9D, HYS+LY). Double immunofluorescence confocal microscopy showed that the HYS-32-induced depletion of EB1 at microtubule plus end was partially recovered by co-treatment of LY294002 in HYS-32-treatedastrocytes (Fig 8, HYS+LY; compare arrowheads and double arrowheads in Enlarged). Treatment of LY294002 alone had little effect on the distance between microtubule tips and the cell border (Fig 9D, LY) and EB1 distribution (Fig 8, LY) in astrocytes.

### SB415286 Partially Reverses the HYS-32-induced Inhibition of Astrocyte Migration

**Fig 10:**
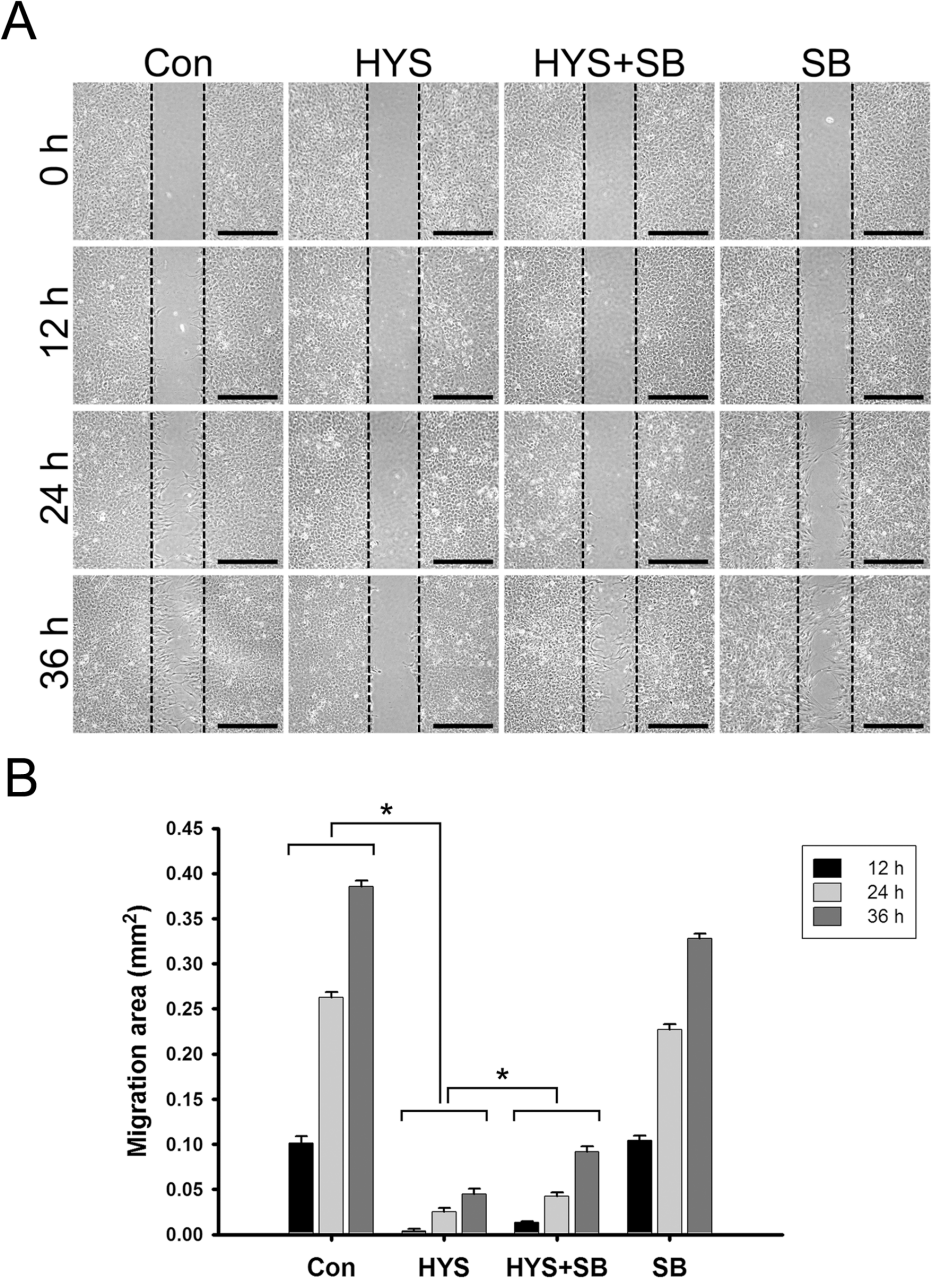
SB415286 partially reverses the HYS-32-inhibited astrocyte migration. (A) Control astrocytes (Con) or astrocytes treated with 5 μM HYS-32 (HYS), co-treated for 24 h with 5 μM HYS-32 and 20 μM SB415286 (HYS+SB), or treated with 20 μM SB415286 (SB) were analyzed with migration assay culture inserts 0, 12, 24, and 36 h after treatment (bars = 500 μm). (B) The data for the astrocytes migration area were collected from three independent experiments. *p<0.01 compared to HYS using one-way ANOVA with Dunnett’s post-hoc test. The dotted lines marked the migration starting edge.

To verify the effect of HYS-32 on astrocytes’ migration ability, we employed an *in vitro* wound healing model. The approach involves creating a wound on a confluent monolayer of primary rat astrocytes using a commercial apparatus as described in **Materials and Methods**. Migration ability was then quantitated based on the closed-wound area as cells moved into the inflicted void. We observed that the control astrocytes began extending their processes into the wound area at 12 h, and slowly migrated to the center area at 24 h and 36 h. The wound width was approximately 500 μm, which was closed in 36 to 48 h in control astrocytes (Fig 10A, Con). HYS-32 nearly inhibited the entire migration of treated astrocytes from 12 h and up to 36 h (Fig 10A and 10B, HYS). Co-treatment with SB415286 only partially rescued the HYS-32-impeded cell migration (Fig 10A and 10B, HYS+SB) and failed to restore normal astrocyte migration. Treatment of SB415286 alone had little effect on astrocyte migration (Fig 10A and 10B, SB). Further, removal of HYS-32 was performed to confirm the role of HYS-32 on astrocyte migration. In comparison with prolonged HYS-32 treatment, HYS-32 removal dramatically restored astrocyte motility, resulting in nearly half of the original wound area coverage (S6 Fig).

## Discussion

Mimori-Kiyosue and Tsukita proposed a model wherethe instability of microtubule dynamics with inter-conversion between shortening and growth of the plus-end contributes to a “search-and-capture” process for cell shape, mitosis, and migration through +TIPs [24]. EB1 knockdown by small interfering RNA caused another +TIP, cytoplasmic linker protein-170 (CLIP-170), to dislodge from microtubule plus ends, leading to the disruption of cell polarity and microtubule dynamics [25]. When EB1 was mutated by a truncation of its N-terminus, rescue of microtubule growth failed [26]. The effect of EB1 on microtubule dynamics was also shown in another study using RNAi, indicating that a loss of EB1 at the plus end suppresses microtubule growth [27]. Furthermore, a study using fluorescent time-lapse video microscopy demonstrated that patupilone, a microtubule stabilizing agent, reduces EB1 accumulation at the microtubule plus ends, thereby resulting in an increased rate of microtubule catastrophes [9]. Interestingly, we showed that treatment of HYS-32 eliminates EB1 distribution from the MT plus ends and prevents the microtubule from targeting the cell cortex in rat astrocytes without altering EB1 levels. Additionally, the dissociation of EB1 from microtubule plus ends began 2 h after HYS-32 treatment followed by a significant shrinkage of microtubule at 6 h after HYS-32 treatment. Since EB1 dislodgement from microtubule plus ends was observed earlier than the microtubule catastrophes, we speculate that HYS-32 may prevent the microtubule from projecting to the cell border by reducing the accessibility of EB1 to tubulin subunits at microtubule plus ends. Furthermore, our study demonstrated that removal of HYS-32 restored EB1 comet-like streaks and reorganized the microtubule distribution at the cell cortex, implying that HYS-32-inducedmicrotubule catastrophes and depletion of EB1 comet-like streaks at microtubule plus ends are reversible. Together these results indicate that HYS-32 could modulate EB1-microtubule interaction at microtubule plus ends to regulate microtubule dynamics at the cell cortex.

Among the abundant regulatory factors, serine/threonine kinase GSK3β is especially important for delivering upstream signals essential for modulating cell polarity and microtubule dynamic instability [28]. GSK3β activity is significantly reduced by phosphorylation at Ser-9 residue and facilitated by phosphorylation at Tyr-216 residue. In hippocampal neurons, activation of the PI3K-Akt/PKB signaling pathway caused phosphorylation of GSK3β at S9, which may result in GSK3β deactivation [29,30]. In this study, our results showed that dissociation of EB1 from microtubule plus ends was concurrent with an increase in the levels of GSK3β-pY216 and GSK3β-pS9 after HYS-32 treatment. Furthermore, GSK3β inhibitor SB415286 significantly inhibited HYS-32-induced GSK3β-pY216 phosphorylation, which could partially restore EB1 distribution on microtubule plus ends and attenuate microtubule catastrophes. PI3K inhibitor LY294002 also inhibited the HYS-32-induced GSK3β-pS9 phosphorylation and partially restored EB1 distribution on microtubule plus ends. To our surprise, LY294002 further inhibited the HYS-32-induced GSK3β-pY216 phosphorylation indicating a possible cross-talk between PI3k and GSK3β signaling pathways in regulating microtubule dynamic instability in astrocytes. LY294002 is known to have PI3K-independent effects on other signaling mechanisms [31]. However, our results showed that treatment of LY294002 alone had no effect on total GSK3β protein levels, thus ruling out a possible direct effect on expression of GSK3β. All together, these results imply that the HYS-32-induced dissociation of EB1 in microtubule plus ends is mediated through the PI3K-GSK3β signaling mechanism. Our confocal microscopy revealed that HYS-32-induced microtubule catastrophes and EB1 depletion at microtubule plus ends both failed to be completely reversed by GSK3β inhibitor SB415286 or PI3K inhibitor LY294002, implying that other regulatory mechanisms may also participate in the microtubule catastrophes.

Previous studies have shown that the lattice-binding and the plus end-binding activities between the +TIP protein CLASP2 and microtubules depend on the regulation of the GSK3β and distinct domains of CLASP2 [32,33]. Increased or decreased activity of the GSK3β can respectively cause the dissociation or stabilization of the EB1-microtubule interaction [33]. However, another study showed an opposite pattern in adult DRG neurons where CLASP2 disassociates from microtubule plus ends at high GSK3β activity and the binding of CLASP2 to microtubule lattices increased at low GSK3β activity [34]. In the present study, the most significant microtubule changes were seen after HYS-32 treatment for 24 h when the inactive form of GSK3β-pS9 was most highly expressed. Our time course experiment showed that the GSK3β phosphorylation state gradually switched from GSK3β-pY216 to GSK3β-pS9 and the ratio of Y216/S9 decreased from 1.8 to 0.7 after HYS-32 treatment for 2–24 h, suggesting the activation level of the GSK3β changed from a higher GSK3β activity to a lower GSK3β activity. These temporal changes in the Y216/S9 ratio is reversible as our inhibitor studies showed that it first dropped to 0.75 after HYS-32 treatment for24 h, and returned to 0.91 during the HYS-32 and LY294002 co-treatment, suggesting that HYS-32 switches GSK3β to a lower activity state via a PI3K-mediated pathway. Using confocal microscopy and co-immunoprecipitation analysis, we further demonstrated that short-term treatment of astrocytes with HYS-32 for 2–6 h eliminates EB1 distribution from the microtubule plus ends and causes a slight decrease in the association between EB-1 and β-tubulin by up-regulating GSK3β-pY216 phosphorylation. However, an extensive distribution of EB1 on the microtubule lattices and an increase in the association between EB-1 and β-tubulin by up-regulating GSK3β-pS9 phosphorylation were observed after prolonged treatment of HYS-32 for 24 h while EB1 protein levels remained unchanged. Collectively, these results suggest that the HYS-induced unbalance of GSK3β activities may affect stability of EB1 and its association with microtubule plus ends, which may eventually lead to microtubule catastrophes.

In a study using electron microscopy, the expression of abundant microtubules in reactive astrocytes has been suggested to be the main cause of glial scar formation and myelin sheath degeneration [35]. In agreement with these findings, increased immunoreactivity of tubulin was also observed by confocal immunofluorescence microscopy in reactive astrocytes in the immediate vicinity of a destructive lesion [36]. These results imply that abnormal microtubule dynamics are associated with change in cell shape of astrocytes, which is reversed by colchicine treatment [37]. Decreased expression of N-cadherin has been suggested to alter cell polarity and speed and directionality of glial cell migration [38]. However, HYS-32-induced microtubule coiling did not appear to alter cell shape as F-actin and N-cadherin were not affected. Microtubule dynamic instability induced by microtubule-targeting agents has been suggested to suppress multiple cellular processes including migration [39,40]. Our present study revealed that HYS-32 treatment inhibited rat astrocyte migration following microtubule catastrophe and EB1 dissociation from microtubule plus ends. In agreement with this context, microtubule-targeting agents affect cell migration through a loss of EB1 accumulation at microtubule plus ends and microtubule stabilization at cell cortex [41]. Thus, maintenance of EB1 on microtubule plus ends at the cell cortex is required for astrocytes to facilitate cell migration.

**Fig 11:**
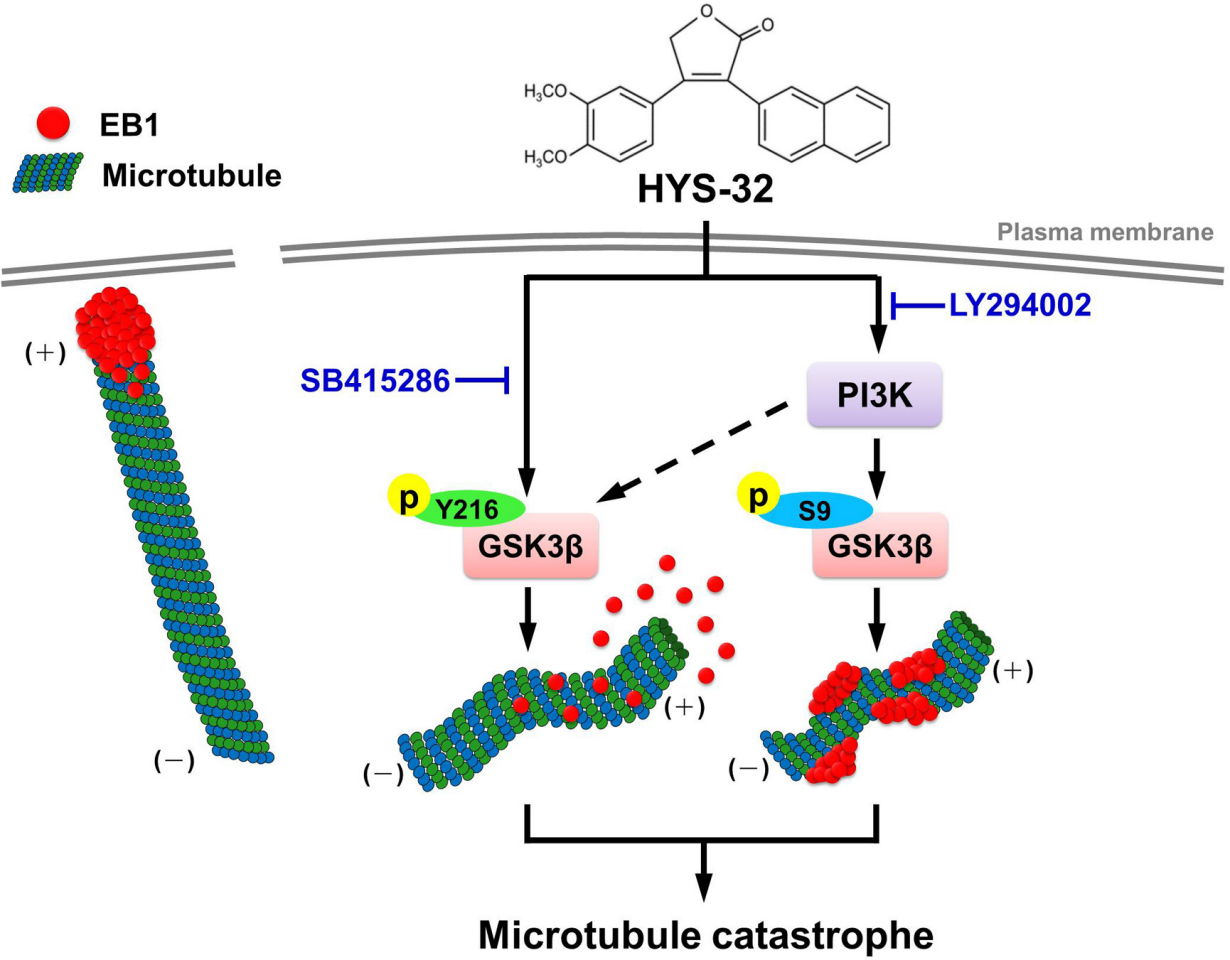
Illustration of the signaling pathway involved in the effect of HYS-32 on microtubule catastrophes in rat astrocytes. HYS-32 induces microtubule catastrophes by causing EB1 dislodgement from microtubule plus ends and EB1 accumulation on the microtubule lattice through the modulation of the PI3K-GSK3β signaling pathway.

In conclusion, we have demonstrated that a HYS-32-induced microtubule catastrophe causes EB1 dislodgement from microtubule plus ends and EB1 accumulation on the microtubule lattice through the modulation of the PI3K-GSK3β signaling pathway (Fig 11). The novel biological efficacy of HYS-32 on microtubule dynamic instability in astrocytes may present as a new potential therapeutic drug for axonal regeneration around the glial scar following severe CNS injuries.

## Supporting Information

S1 Fig HYS-32 induces microtubule catastrophes in a dose-dependent manner. Control astrocytes (Con) or astrocytes treated for 24 h with different concentrations (0.5, 1, 2, 5, or 10 μM) of HYS-32 were fixed in cold acetone and double-stained for N-cadherin (red) and β-tubulin (green) and subjected to confocal microscopy. Arrowheads indicate the intercellular junctions (bars = 20 μm).
(TIF)

S2 Fig HYS-32 induces microtubule catastrophes in a time-dependent manner. Control astrocytes (Con) or astrocytes treated for various time periods (0.5, 1, 1.5, 2, 4, 6, 12, or 24 h) with 5 μM HYS-32 were fixed in cold acetone and double-stained for N-cadherin (red) and β-tubulin (green) and subjected to confocal microscopy. Arrowheads indicate the intercellular junctions (bars = 20 μm).
(TIF)

S3 Fig HYS-32 has no effect on F-actin organization. Control astrocytes (Con) or astrocytes treated with 5 μM HYS-32 for 24 h (HYS) were fixed in cold acetone and double-stained for β-tubulin (green) and F-actin (blue) and subjected to immunofluorescence microscopy. Images were merged to show co-localization (Merged) (bars = 20 μm).
(TIF)

S4 Fig HYS-32 induces microtubules forming a coiled mass in the perinuclear area. Control astrocytes (Con) or astrocytes treated with 5 μM HYS-32 for 24 h (HYS) were fixed in cold acetone and triple-stained for β-tubulin (green), EB1 (red), and F-actin (blue) and subjected to immunofluorescence microscopy. Images were merged to show co-localization (Merged). Arrowheads indicate microtubule tips at the cell cortex (bars = 20 μm).
(TIF)

S5 Fig HYS-32 treatment does not affect the protein levels of β-tubulin and EB1. (A) Cell lysates from control astrocytes (Con) or astrocytes treated for 2, 6, 12, or 24 h with 5 μM HYS-32 were subjected to 10% SDS-PAGE, and analyzed by immunoblotting with antibodies against β-tubulin, EB1, or GAPDH. (B) Densitometric analyses of β-tubulin and EB1 expressed as the density of the bands in the treated group relative to the control. The results were collected from five independent experiments. *p*>0.05 compared to control using one-way ANOVA with Dunnett’s post-hoc test.
(TIF)

S6 Fig HYS-32 removal facilitates the astrocyte migration. Control astrocytes (Con) or astrocytes treated with 5 μM HYS-32 (HYS) for 0 to 72 h (0 h, 6 h, 24 h, 48 h, or 72 h) or treated for 24 h with 5 μM HYS-32 then replaced with normal culture medium (HYS-R) for 0 to 48 h (R0 h, R24 h, R36 h, or R48 h) in the absence of HYS-32 were analyzed with wound healing assay. The data were collected from three independent experiments. *p<0.01 compared to control astrocytes, ^*#*^ *p*<0.01 compared to HYS-32-treated astrocytes using one-way ANOVA with Dunnett’s post-hoc test.
(TIF)
