# Supplementary figures and images for: HYS-32-Induced Microtubule Catastrophes in Rat Astrocytes Involves the PI3K-GSK3beta Signaling Pathway
Source: PLoS One. 2015 May 4;10(5):e0126217. doi: 10.1371/journal.pone.0126217 (PMC4418738; doi:10.1371/journal.pone.0126217)

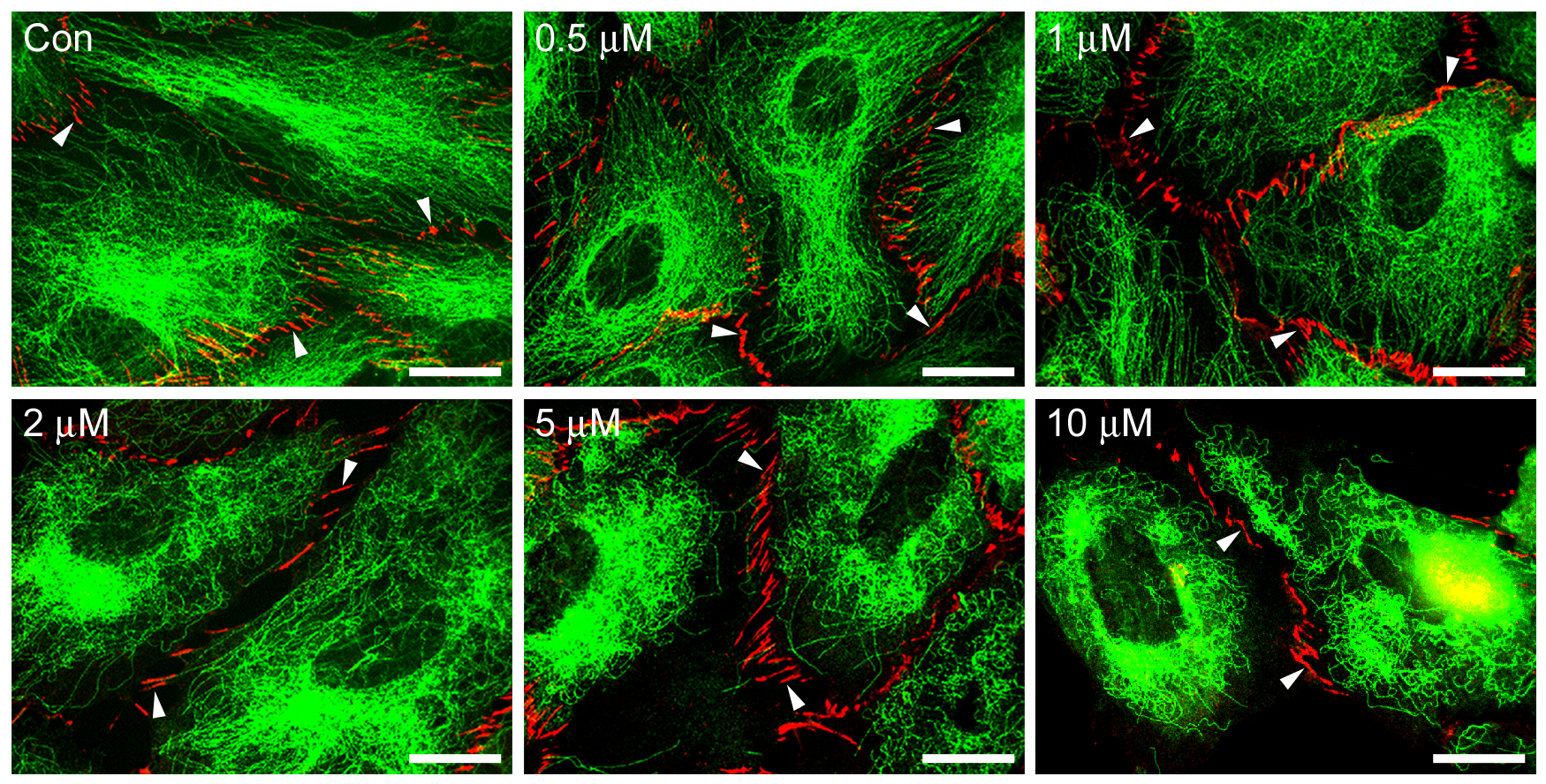

Supplement: S1 Fig — Control astrocytes (Con) or astrocytes treated for 24 h with different concentrations (0.5, 1, 2, 5, or 10 μM) of HYS-32 were fixed in cold acetone and double-stained for N-cadherin (red) and β-tubulin (green) and subjected to confocal microscopy. Arrowheads indicate the intercellular junctions (bars = 20 μm). (TIF) [file pone.0126217.s001.tif]

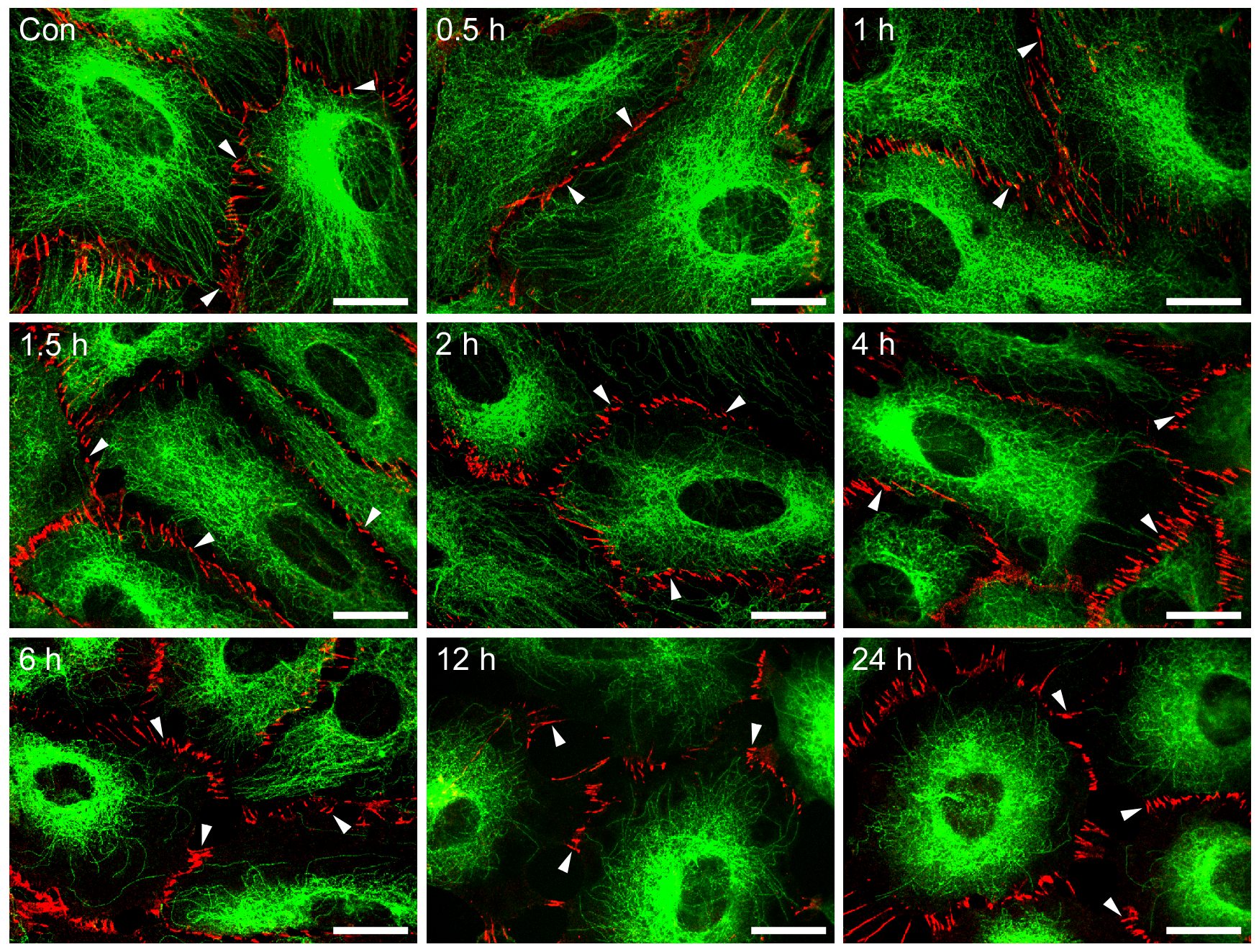

Supplement: S2 Fig — Control astrocytes (Con) or astrocytes treated for various time periods (0.5, 1, 1.5, 2, 4, 6, 12, or 24 h) with 5 μM HYS-32 were fixed in cold acetone and double-stained for N-cadherin (red) and β-tubulin (green) and subjected to confocal microscopy. Arrowheads indicate the intercellular junctions (bars = 20 μm). (TIF) [file pone.0126217.s002.tif]

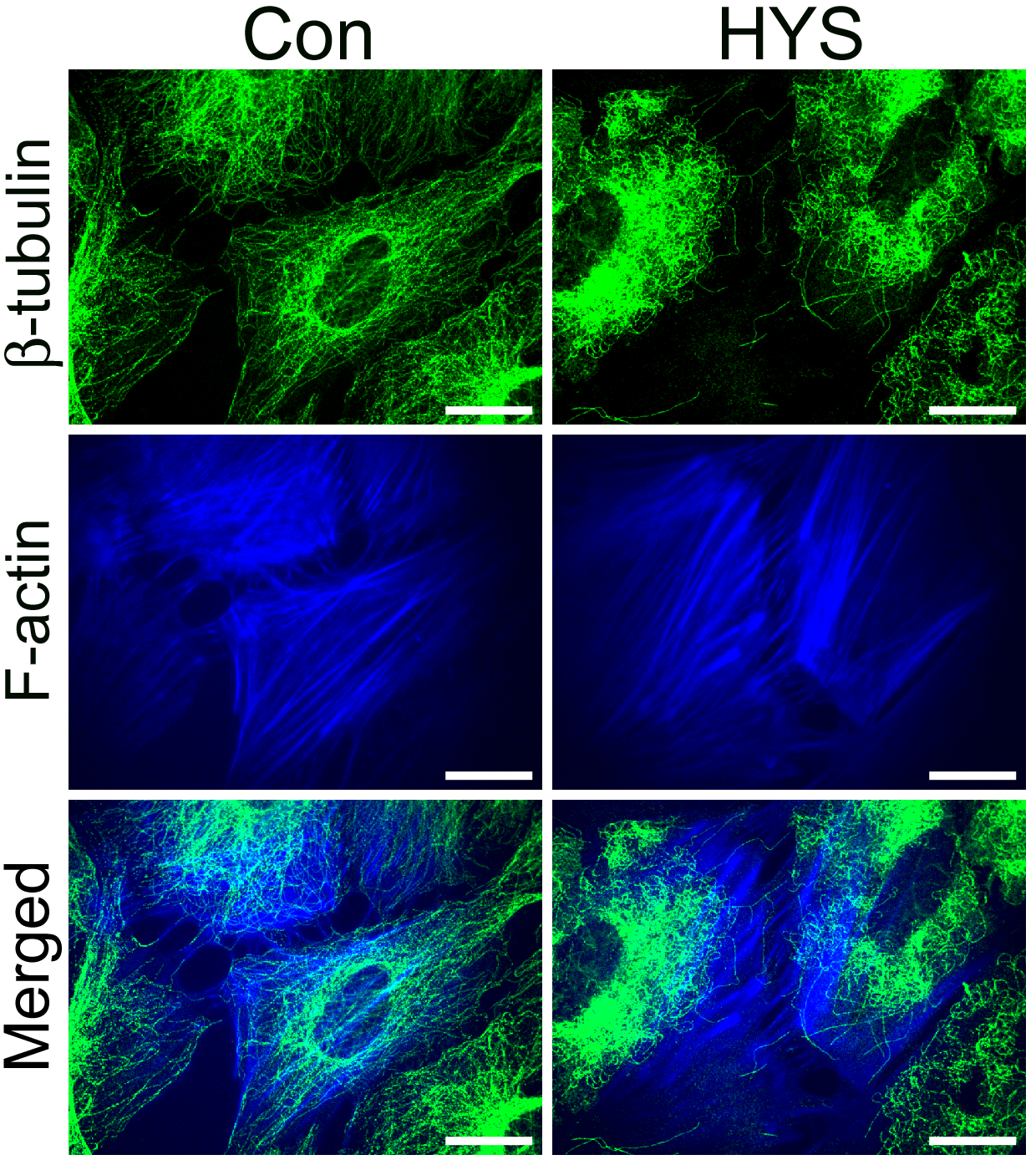

Supplement: S3 Fig — Control astrocytes (Con) or astrocytes treated with 5 μM HYS-32 for 24 h (HYS) were fixed in cold acetone and double-stained for β-tubulin (green) and F-actin (blue) and subjected to immunofluorescence microscopy. Images were merged to show co-localization (Merged) (bars = 20 μm). (TIF) [file pone.0126217.s003.tif]

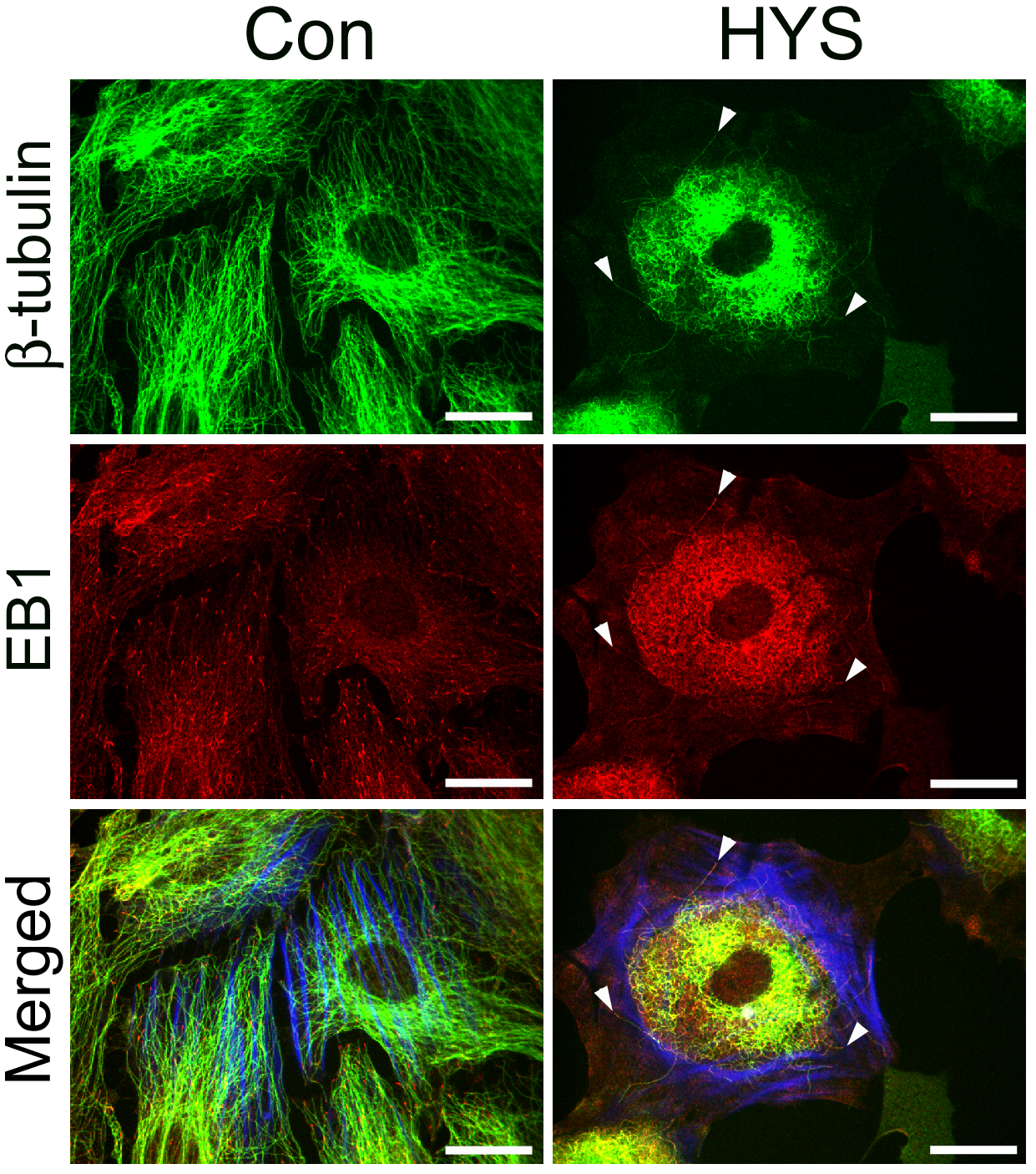

Supplement: S4 Fig — Control astrocytes (Con) or astrocytes treated with 5 μM HYS-32 for 24 h (HYS) were fixed in cold acetone and triple-stained for β-tubulin (green), EB1 (red), and F-actin (blue) and subjected to immunofluorescence microscopy. Images were merged to show co-localization (Merged). Arrowheads indicate microtubule tips at the cell cortex (bars = 20 μm). (TIF) [file pone.0126217.s004.tif]

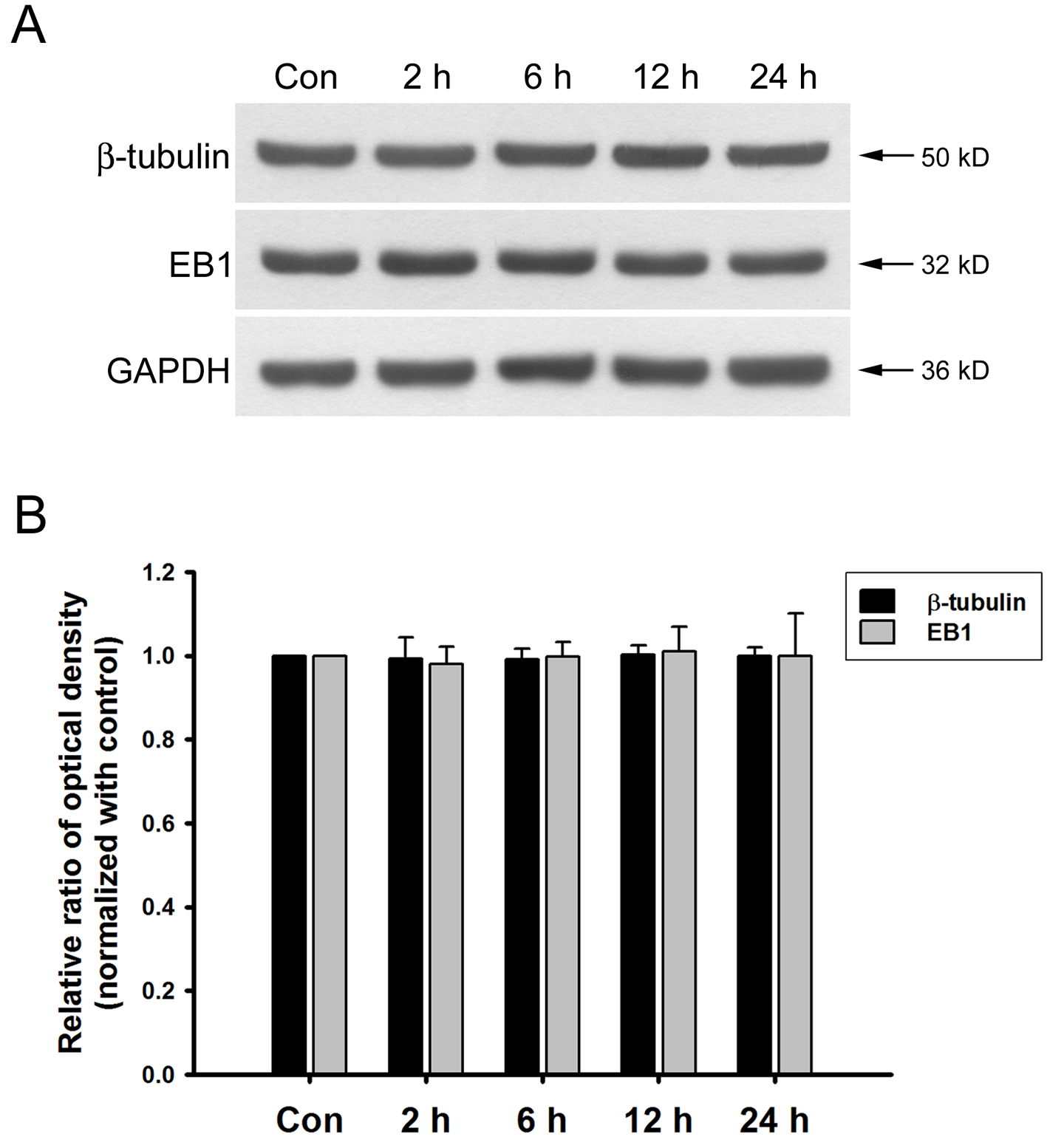

Supplement: S5 Fig — (A) Cell lysates from control astrocytes (Con) or astrocytes treated for 2, 6, 12, or 24 h with 5 μM HYS-32 were subjected to 10% SDS-PAGE, and analyzed by immunoblotting with antibodies against β-tubulin, EB1, or GAPDH. (B) Densitometric analyses of β-tubulin and EB1 expressed as the density of the bands in the treated group relative to the control. The results were collected from five independent experiments. p>0.05 compared to control using one-way ANOVA with Dunnett’s post-hoc test. (TIF) [file pone.0126217.s005.tif]

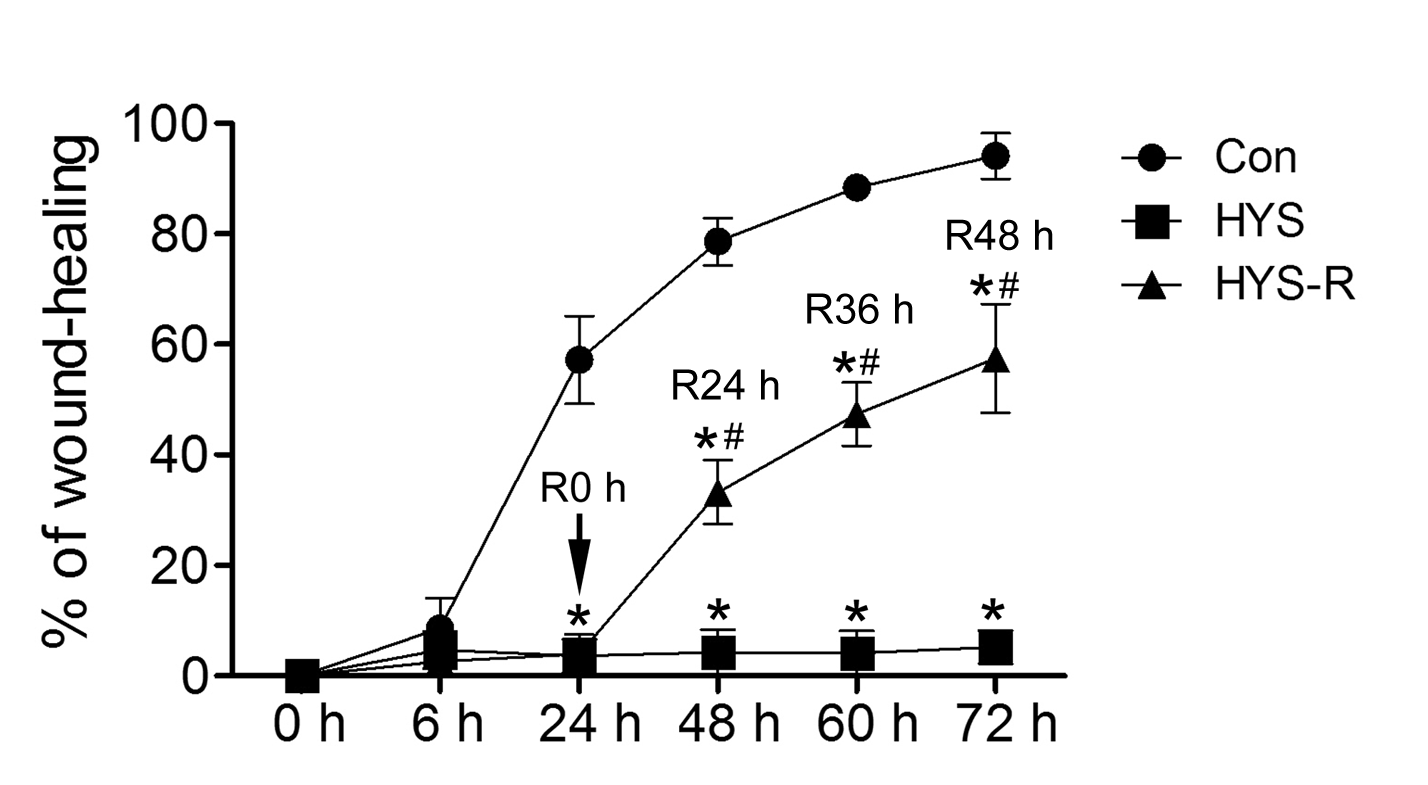

Supplement: S6 Fig — Control astrocytes (Con) or astrocytes treated with 5 μM HYS-32 (HYS) for 0 to 72 h (0 h, 6 h, 24 h, 48 h, or 72 h) or treated for 24 h with 5 μM HYS-32 then replaced with normal culture medium (HYS-R) for 0 to 48 h (R0 h, R24 h, R36 h, or R48 h) in the absence of HYS-32 were analyzed with wound healing assay. The data were collected from three independent experiments. *p<0.01 compared to control astrocytes, # p<0.01 compared to HYS-32-treated astrocytes using one-way ANOVA with Dunnett’s post-hoc test. (TIF) [file pone.0126217.s006.tif]
